# Gene Polymorphisms and Biological Effects of Vitamin D Receptor on Nonalcoholic Fatty Liver Disease Development and Progression

**DOI:** 10.3390/ijms24098288

**Published:** 2023-05-05

**Authors:** Evanthia Tourkochristou, Athanasia Mouzaki, Christos Triantos

**Affiliations:** 1 Division of Gastroenterology, Department of Internal Medicine, Medical School, University of Patras, University Hospital of Patras, Rion, 26504 Patras, Greece; 2 Division of Hematology, Department of Internal Medicine, Medical School, University of Patras, 26504 Patras, Greece

**Keywords:** VDR, polymorphisms, protein, NAFLD, disease

## Abstract

Nonalcoholic fatty liver disease (NAFLD) is the most common chronic liver disease, with increasing prevalence worldwide. The genetic and molecular background of NAFLD pathogenesis is not yet clear. The vitamin D/vitamin D receptor (VDR) axis is significantly associated with the development and progression of NAFLD. Gene polymorphisms may influence the regulation of the VDR gene, although their biological significance remains to be elucidated. VDR gene polymorphisms are associated with the presence and severity of NAFLD, as they may influence the regulation of adipose tissue activity, fibrosis, and hepatocellular carcinoma (HCC) development. Vitamin D binds to the hepatic VDR to exert its biological functions, either by activating VDR transcriptional activity to regulate gene expression associated with inflammation and fibrosis or by inducing intracellular signal transduction through VDR-mediated activation of Ca^2+^ channels. VDR activity has protective and detrimental effects on hepatic steatosis, a characteristic feature of NAFLD. Vitamin D-VDR signaling may control the progression of NAFLD by regulating immune responses, lipotoxicity, and fibrogenesis. Elucidation of the genetic and molecular background of VDR in the pathophysiology of NAFLD will provide new therapeutic targets for this disease through the development of VDR agonists, which already showed promising results in vivo.

## 1. Introduction

NAFLD is the most common chronic liver disease, with a worldwide prevalence of 25% in adults, which continues to rise [1]. In the United States, NAFLD cases are expected to increase from 83.1 million in 2015 to 100.9 million in 2030 [2]. Given the association between NAFLD and a spectrum of severe liver complications, including cirrhosis, HCC, and increased liver-related mortality, disease management and the development of effective therapeutic strategies are critical [3]. NAFLD is a set of diseases caused by fat accumulation in the liver and is closely related to obesity, diabetes, and metabolic syndrome [4]. To date, no approved therapy was developed, and combination treatment is proposed for NAFLD patients. The complex pathophysiology and diversity of NAFLD disease phenotypes complicate the development of effective treatment and patient management, which increases the disease burden [3].

The genetic and molecular background of NAFLD pathogenesis is currently under investigation. The vitamin D/VDR axis is significantly associated with NAFLD pathogenesis and disease progression, considering the role of vitamin D/VDR signaling in regulating inflammation, immune responses, and lipogenic gene expression [5,6]. By binding to VDR, vitamin D exerts its anti-fibrotic, anti-inflammatory, and insulin-sensitizing functions in liver cells and is involved in immune-metabolic signaling pathways within the gut–adipose tissue–liver axis [7]. Polymorphisms in the VDR gene were also associated with the severity of liver fibrosis in patients with biopsy-proven NAFLD [8] and with liver enzyme activity in NAFLD patients treated with calcitriol [9]. An association between VDR gene polymorphisms and NAFLD-related liver complications, including steatosis, cirrhosis, and HCC, was reported [10,11,12]. It is possible that VDR plays a genetic and biological role in the pathophysiology of NAFLD that remains to be elucidated. 

In this review, we provide an update on the combined effect of gene polymorphisms in the VDR gene and VDR signaling in the development and progression of NAFLD, which will contribute to the exploration of molecular therapeutic targets for the prevention of NAFLD-related disease complications and the effective treatment of patients. 

## 2. Methodology

A comprehensive literature search was conducted on the role of gene polymorphisms and biological mechanisms of VDR in the development and progression of nonalcoholic fatty liver disease. The PubMed database was used for the literature search with the following search strategies and keywords: (1) (gene polymorphisms) AND (vitamin D receptor) OR (VDR) AND (NAFLD), (2) (VDR gene polymorphisms) AND (NAFLD development), (3) (VDR gene polymorphisms) AND (NAFLD progression), (4) (VDR gene polymorphisms) AND (NASH) AND (liver fibrosis) OR (liver cirrhosis) AND (hepatocellular carcinoma) OR (HCC), (5) (vitamin D receptor) OR (VDR) AND (NAFLD development), (6) (vitamin D receptor) OR (VDR) AND (NAFLD progression), (7) (vitamin D receptor) OR (VDR) AND (vitamin D) AND (NAFLD), and (8) (vitamin D receptor) OR (VDR) AND (NASH) AND (liver fibrosis) OR (liver cirrhosis) AND (hepatocellular carcinoma) OR (HCC), including all English language articles published between January 1990 and March 2023. Electronic and manual searches were performed, focusing on the role of VDR gene polymorphisms and VDR biological effects on the development and progression of NAFLD to NASH, fibrosis/liver cirrhosis, and HCC.

## 3. Genetics and Biology of VDR Gene Polymorphisms

### 3.1. Polymorphisms of the VDR Gene

VDR is a nuclear transcription factor that binds to hormonally active vitamin D [1,25(OH)_2_D_3_] and mediates its biological function. The importance of the VDR molecule as a target for the study of underlying mechanisms in disease pathophysiology is highlighted by the VDR-mediated regulation of several genes. The VDR–vitamin D complex enters the nucleus and regulates the expression of more than 900 genes [13,14]. Genetic variants can affect VDR gene regulation and impact VDR gene expression and function. Polymorphisms in the primary VDR promoter (G-1739A: rs11568820, A-1012G: rs4516035) can alter transcription factor binding for Cdx-2 and GATA [15,16]. Specifically, the VDR binding capacity for Cdx-2 and GATA factors was increased in the presence of the A nucleotide alleles of G-1739A and A-1012G compared to their allelic counterparts. Considering that Cdx-2 is tissue-specific, it might affect VDR expression in certain cell types in response to environmental stimuli [16]. FokI (rs2228570) is a single nucleotide polymorphism (SNP) in the translation initiation codon of the VDR gene characterized by an allelic transition from T(f) to C(F). The presence of the F allele in the VDR gene results in a VDR protein that is three amino acids shorter and has a higher transactivation capacity than the non-truncated protein [17]. In the last decade, studies examining gene polymorphisms in the VDR gene focused on the role of VDR SNPs in regulating gene expression and protein production to interpret the observed differential disease susceptibility associated with SNPs [18]. ApaI (rs7975232), BsmI (rs1544410), TaqI (rs731236), and FokI (rs2228570) are the most studied SNPs in the VDR gene. EcoRV (rs4516035) and Tru9I (rs757343) are two other common restriction fragment length polymorphisms (RFLPs) found in intron 8 at the 3’ end of the VDR [19,20]. The FokI SNP in the VDR promoter can affect VDR expression, as reported above. ApaI, BsmI, and TaqI are located in the 3’UTR of the VDR gene and were suggested to play a role in regulating the VDR gene, as shown by the luciferase activity of these SNP variants in COS-7 cells [19]. Although there is no evidence for the functional effects of ApaI, BsmI, and TaqI, as they are noncoding/silent SNPs, a role of these SNPs in VDR mRNA stability was suggested [16]. 

In the case of noncoding SNPs, it was also speculated that the presence of linkage disequilibrium (LD) between silent SNPs and other functional variants may explain the function of silent SNPs [18]. The presence of the ‘B’ allele of BsmI and the ‘t’ allele of TaqI in healthy individuals was associated with lower VDR protein levels, whereas the TT genotype of TaqI was associated with higher VDR protein levels [21]. The TaqI SNP at a CpG site also affects the methylation of CGI 1060 at the 3’-end of the VDR, indicating a possible effect of VDR SNPs on the epigenetic regulation of the VDR [22]. Other polymorphisms were found in the promoter region, in and around exons 1f-1c, 2-9, and in the 3’-UTR, with most reported in the regulatory regions. It seems likely that most gene polymorphisms and population variance occur in the gene regions (e.g., 5’-promoter region, 3’-UTR) that regulate the level of VDR gene expression. Polymorphisms in the protein sequence could have significant functional implications, such as changes in ligand affinity or binding to DNA [23].

### 3.2. Biological Significance of VDR Gene Polymorphisms

The complex organization of the VDR gene makes identification of functional polymorphisms difficult, and most polymorphisms that were used to interpret association studies are anonymous. Any observed association can likely be explained by assuming a LD between a truly functional sequence variation elsewhere in the gene and an allele of the anonymous polymorphism [23]. Overall, most polymorphisms in the VDR gene are widespread in the population, and their effects on VDR function remain to be elucidated. Determining the presence of polymorphisms in specific candidate gene regions will help to understand the genetic and possible functional relationship between the different polymorphisms. Examination of the LD between polymorphisms will provide insight into how specific combinations of alleles in the candidate gene influence the amplification or attenuation of specific effects on gene function. 

**Figure 1:**
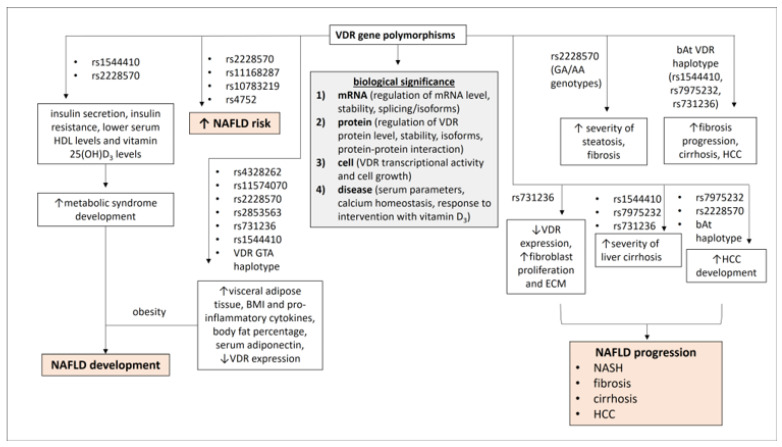
The role of VDR gene polymorphisms in the development and progression of NAFLD. The biological significance of VDR gene polymorphisms is highlighted at four levels (mRNA, protein, cell, and disease). Certain VDR gene polymorphisms could promote the development of NAFLD by being involved in the development of metabolic syndrome and adipose tissue activity, and they could influence the progression of NAFLD to more severe phenotypes by increasing steatosis, fibrosis, severity of cirrhosis, and development of HCC. ↑: increase, ↓: decrease.

In recent years, studies analyzed various bioresponse parameters, using the anonymous polymorphisms BsmI, Bsm-Apa-Taq haplotypes, the polyA VNTR in the 3’-UTR, in vitro biological and molecular systems, and in vivo measurements of biochemical markers, as well as the response to vitamin D, calcium treatment, and hormone replacement therapy [23]. According to these studies, there are four different levels of organization in physiology that can simultaneously determine the functionality of VDR polymorphisms and the association between VDR polymorphisms and diseases in epidemiological studies. The mRNA level refers to the effects of VDR polymorphisms on the regulation of mRNA levels, stability, and splicing/isoforms. The protein level refers to the effects of VDR polymorphisms on the regulation of VDR protein levels, stability, isoforms, and protein–protein interaction. The cellular level refers to the effects of VDR polymorphisms on transcriptional activity and cell growth, and the human level links VDR polymorphisms to serum parameters, calcium homeostasis, and response to intervention with vitamin D_3_ [23] (Figure 1). Investigation of the effects of VDR polymorphisms on vitamin D-VDR signaling in the context of various diseases is ongoing and should provide useful clues for research in personalized medicine and pharmacogenomics.

## 4. VDR Gene Polymorphisms in the Development of NAFLD 

NAFLD is a metabolic fatty liver disease mainly caused by obesity, diabetes type II, dyslipidemia, and insulin resistance [24]. A systematic review analyzing studies on vitamin D-related genetic variations and NAFLD showed that VDR gene polymorphisms rs2228570, rs11168287, rs10783219, and rs4752 were associated with the presence of NAFLD, while NAFLD severity was associated with VDR variants rs2228570 and rs4334089 [25]. VDR SNPs were associated with liver function in NAFLD patients. Calcitriol-treated patients with NAFLD and the Ff genotype of FokI SNP showed a significant decrease in alkaline phosphatase activity, which is a direct parameter of response to vitamin D treatment [9].

The development of NAFLD is closely related to metabolic syndrome, as about 90% of NAFLD patients have features of metabolic syndrome [26]. An important interaction between VDR gene polymorphisms and metabolic syndrome components was established. In particular, the VDR gene polymorphisms BsmI (rs1544410) and FokI (rs2228570) were suggested to influence insulin secretion and insulin resistance. Individuals with metabolic syndrome who were heterozygous for the FokI SNP had higher iPTH levels (a marker for induction of insulin secretion) and higher β cell secretion (HOMA β) than individuals without this polymorphism. Individuals carrying the mutant recessive homozygous ff genotype had a significantly higher insulin resistance index (HOMA-IR) compared to the heterozygous Ff genotype. The presence of the mutant bb genotype of the BsmI SNP was associated with lower serum 25(OH)D_3_ levels, which is considered to be an indirect effect of BsmI on the metabolic syndrome, given the role of vitamin D in regulating insulin secretion and sensitivity [27].

Obesity is associated with an increased risk of developing NAFLD [28]. Obesity is characterized by excessive fat accumulation in adipose tissue, which can impair fatty acid metabolism. Steatosis, a characteristic feature of NAFLD, may develop from an imbalance between fatty acid uptake/synthesis and fatty acid oxidation/secretion [28]. A possible role of VDR gene polymorphisms in regulating adipose tissue activity was demonstrated. The G allele of VDR SNP rs4328262 was associated with an increase in visceral adipose tissue, the A allele of VDR SNP rs11574070 with body fat percentage, and the A allele of VDR SNP rs2228570 and the T allele of VDR SNP rs2853563 with serum adiponectin concentration in adult subjects [10]. The VDR alleles (G) rs731236 (TaqI) and (T) rs1544410 (BsmI) might increase the risk of obesity, as they were associated with higher body mass index values in obese individuals. The VDR GTA haplotype, composed of the VDR SNPs rs731236 (G)/rs1544410 (T)/rs7975232 (A), was also associated with upregulation of inflammasome components, increased levels of proinflammatory cytokines, and lower VDR expression [29].

Development of fatty liver disease was reported after liver transplantation. Genotype analysis of selective graft biopsies from liver tissue after living donor liver transplantation was performed to investigate the effects of genetic variants in the vitamin D-VDR system on vitamin D maintenance after living donor liver transplantation [30]. NAFLD was significantly associated with low serum vitamin D levels and progression of liver fibrosis [31,32]. The presence of the VDR polymorphism rs2228570 was shown to be related to low serum vitamin D levels and to influence the development of fatty liver disease in recipients after living donor transplantation [30] (Figure 1).

## 5. VDR Gene Polymorphisms in NAFLD Disease Progression

NAFLD is characterized by an accumulation of triglycerides in hepatocytes that exceeds 5% of liver weight and can progress to various disease phenotypes, from simple steatosis to nonalcoholic steatohepatitis (NASH), which can lead to fibrosis, cirrhosis, and HCC [33]. Considering the increasing obesity in Western countries, NAFLD and NASH became the most common forms of chronic liver disease [34,35,36]. The progression of chronic liver disease was associated with VDR gene polymorphisms. In particular, the combination of the rs1544410 (BsmI) C, rs7975232 (ApaI) C, and rs731236 (TaqI) A alleles in the NR1I1 CCA (bAt) haplotype was significantly associated with the progression of fibrosis and the development of cirrhosis and HCC in HCV-infected patients who were diagnosed with alcohol-induced liver injury [37,38,39]. The bAt haplotype was also associated with an increased risk of chronic liver disease and HCC according to ethnicity. Genetic polymorphisms in the patatin-like phospholipase-3 (PNPLA3) and VDR genes, which were associated with the risk of liver steatosis, fibrosis, cirrhosis, and liver cancer, were shown to be significantly higher in Native Americans and significantly lower in South American populations of non-Native American origin [40]. 

The initial stage of NAFLD is steatosis. Steatosis may be influenced by VDR SNPs, as the presence of GA/AA genotypes of the FokI SNP was associated with increased severity of steatosis in pediatric patients with NAFLD in the United Kingdom [41]. Excessive fat accumulation in the liver determines the next stage of NASH, which triggers inflammation, leading to increased immune cell infiltration and cytokine secretion. Under the influence of sustained immune activation, hepatic stellate cells (HSCs) are activated and transdifferentiate into myofibroblasts, which produce collagen and promote fibrosis development [42]. Given the observed association between the CC genotype of the BsmI SNP and the advanced fibrosis stage in NAFLD patients, VDR SNPs may influence the progression of NAFLD to fibrosis. NAFLD patients with the CC genotype of BsmI SNP and low serum vitamin D levels had more severe fibrosis compared with other genotypes [8]. The bAt VDR haplotype, which includes the VDR SNPs BsmI rs1544410 C, ApaI rs7975232 A, and TaqI rs731236 A, was associated with rapid fibrosis progression in chronic HCV patients and was proposed as an independent risk for fibrosis progression [38]. FokI rs2228570 TT/TC genotypes were also reported as risk factors for advanced fibrosis in HCV patients [43]. The effect of VDR SNPs on fibrosis development was described in in vitro experiments, which showed that the presence of TaqI SNP (CC genotype) in intestinal fibroblasts was significantly associated with decreased VDR expression, resulting in increased fibroblast proliferation. TaqI SNP was associated with increased expression of extracellular matrix proteins [44] (Figure 1).

Persistent and progressive fibrosis leads to the development of cirrhosis in NAFLD [42]. The VDR SNPs ApaI, BsmI, and TaqI were associated with the severity of cirrhosis [11]. There are no data on the association between VDR gene polymorphisms and the risk of cirrhosis. Most studies showed an association between VDR gene polymorphisms and the development of cirrhosis by describing their effects on fibrosis. The presence of VDR gene polymorphisms was significantly associated with the development of primary biliary cholangitis [45,46]. NAFLD-related cirrhosis may lead to the development of HCC [3]. However, the development of HCC in non-cirrhotic NAFLD was also reported [47]. The VDR SNPs ApaI and FokI may increase susceptibility to HCC in certain populations [48]. The CC genotype of the ApaI SNP and the bAt haplotype were reported to be important factors in the development of HCC in HCV patients [49]. Polymorphisms of the VDR gene were associated with the development of HCC in patients with liver cirrhosis [37] (Figure 1).

## 6. Vitamin D-VDR Signaling in Cells

Vitamin D is a secosteroid hormone consisting of two compounds, vitamin D_2_ and vitamin D_3_. Vitamin D_2_ is a derivative from food and chemical synthesis, while vitamin D_3_ is mainly formed by skin synthesis. Previtamin D_3_ is formed by photochemical conversion of 7-dehydrocholesterol under sunlight in the skin. Thermal isomerization converts previtamin D_3_ to vitamin D_3_, which binds to the vitamin D binding protein (DBP) and is transported to the liver. Both D_2_ and D_3_ are hydroxylated in the liver to 25-hydroxyvitamin D [25(OH)D], which binds to DBP and is excreted into the bloodstream. In the kidney, [25(OH)D] is further hydroxylated to 1,25-dihydroxyvitamin D_3_ [1,25(OH)_2_D_3_], the active form of vitamin D (calcitriol). Next, 1,25(OH)_2_D_3_ is excreted back into the bloodstream and bound to DBP until it enters a target cell [50]. Once 1,25(OH)_2_D_3_ enters the cell, it binds to VDR, which mediates the biological effects of vitamin D. Although VDR is not expressed or is expressed at low levels in hepatocytes, it is highly expressed in non-parenchymal cells, including Kupffer cells, HSCs, and sinusoidal endothelial cells [51]. 

**Figure 2:**
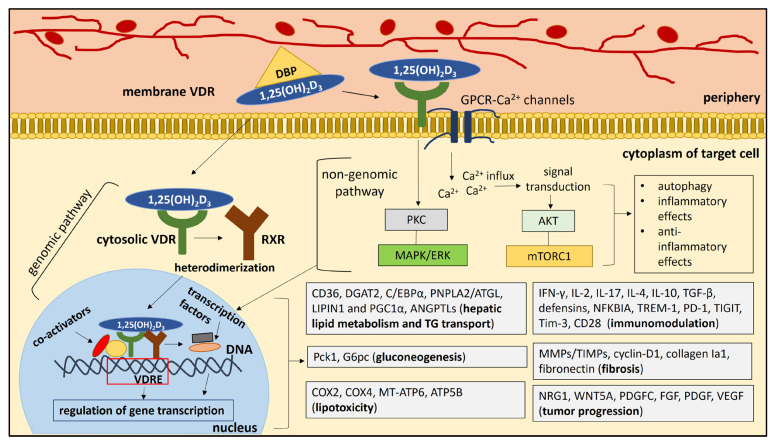
The biological effects of vitamin D-VDR signaling on the target cell. Vitamin 1,25(OH)_2_D_3_ can act through two pathways: the genomic pathway and the nongenomic pathway. The genomic pathway involves the binding of vitamin 1,25(OH)_2_D_3_ to the cytosolic VDR, activating the VDR, which forms a heterodimer with the retinoid X receptor (RXR). The vitamin D_3_-VDR-RXR complex enters the nucleus, where it binds to vitamin D response elements (VDREs) and regulates gene transcription. Binding of VDR-RXR to VDREs can stimulate the recruitment of co-activator proteins to the VDR-RXR complex. The nongenomic pathway refers to the binding of 1,25(OH)_2_D_3_ to membrane VDR, which activates GPCR-mediated calcium channels and initiates Ca^2+^ influx into the cell. Calcium ions act as messengers that mediate intracellular processes of signal transduction. Ca^2+^ activity can trigger downstream signaling pathways, including activation of the AKT pathway and mTORC1 signaling, as well as autophagy, which can exert inflammatory and anti-inflammatory effects. Activation of the PKC and ERK/MAPK signaling pathways is also mediated by the binding of vitamin D to membrane VDR, which may have pro- or anti-inflammatory effects, depending on additional stimuli. Activation of PKC, ERK/MAPK, AKT, and mTORC1 signaling pathways may lead to the activation of transcription factors in the nucleus, which may also interact with VDR and regulate gene transcription. Genomic and non-genomic signaling pathways lead to the regulation of gene expression related to hepatic lipid metabolism/TG transport, gluconeogenesis, lipotoxicity, immune modulation, fibrosis, and tumor progression.

Vitamin 1,25(OH)_2_D_3_ may exert its biological effects on the liver via the genomic and nongenomic pathways. The genomic pathway involves the binding of vitamin 1,25(OH)_2_D_3_ to the cytosolic VDR, which activates the VDR that forms a heterodimer with the retinoid X receptor (RXR). The vitamin D_3_-VDR-RXR complex enters the nucleus, where it binds to the vitamin D response elements (VDREs) in the promoter of target genes (e.g., pro- and anti-inflammatory genes) to induce or repress gene transcription [52]. Binding of VDR-RXR to VDREs can stimulate the recruitment of co-activator proteins to the VDR-RXR complex [53] (Figure 2). A mediator co-activator complex is located between VDRE and RNA polymerase II and initiation complex proteins in the TATA box region. Histone acetyl transferases (HAT) are recruited to the gene by Src coactivators, which can bind to VDR in the presence of 1,25(OH)_2_D_3_ and promote the function of the transcription machinery. A complex of proteins and repressors can also regulate VDR activity in repressing gene transcription [53]. The nongenomic pathway refers to the binding of 1,25(OH)_2_D_3_ to the VDR membrane, which activates GPCR-mediated calcium (Ca^2+^) channels and initiates Ca^2+^ influx into the cell [54]. Calcium ions act as messengers that mediate intracellular processes of signal transduction [55]. Ca^2+^ activity can induce downstream signaling pathways, including activation of the AKT pathway and mTORC1 signaling, as well as autophagy, which can exert inflammatory and anti-inflammatory effects [54]. Activation of the PKC and ERK/MAPK signaling pathways is also mediated by the binding of vitamin D to the membrane VDR, which may lead to the production of pro- or anti-inflammatory mediators, depending on additional stimuli [54] (Figure 2). Bikle [53] proposed a more detailed description of the nongenomic effects of vitamin D. Binding of vitamin D to the membrane activates the VDR-G protein. There is a GTP shift of GDP and a dissociation of the β- and γ-subunits from the now active α-subunit. Phospholipase C (PLC) (β or γ) is activated by Ga-GTP and hydrolyzes phosphatidylinositol bisphosphate (PIP2) to inositol trisphosphate (IP3) and diacylglycerol (DG). DG activates protein kinase C (PKC). IP3 and DG activate intracellular calcium release via the IP3 receptor and PKC, respectively, as second messengers.

## 7. The Role of Vitamin D-VDR Signaling in the Development of NAFLD

NAFLD is a chronic liver disease characterized by the inability of the liver to efficiently metabolize fatty acids, leading to the accumulation of toxic lipid species [56]. The main pathological pathway leading to the development of NAFLD relates to an existing imbalance between the import and export of fat to and from the liver, resulting in an increased influx of free fatty acids (FFAs) and accumulation of triglycerides (TGs) in hepatocytes. The excessive liver fat leads to a lipotoxic milieu in hepatocytes, which can disrupt normal lipid homeostasis [57]. The development of NAFLD is strongly associated with insulin resistance and components of the metabolic syndrome, including obesity and type 2 diabetes [58]. The release of FFAs from subcutaneous adipose tissue and lipolysis of visceral adipose tissue TG are the main source of FFAs in the liver, which are delivered via the bloodstream [28]. Hepatic de novo lipogenesis contributes to liver fat to a lesser extent because TGs are synthesized in hepatocytes from dietary carbohydrates [59]. Simple steatosis results from the complex interplay of hepatic fatty acid uptake, hepatic de novo lipogenesis, fatty acid oxidation, and VDL-mediated fatty acid export [28].

### 7.1. Direct Effects of VDR on the Development of NAFLD

The role of VDR in the development of hepatic steatosis was reported in vivo. Specifically, VDR was shown to regulate hepatic lipid accumulation in adipocytes of female mice fed a low-fat diet. In VDR knock-out (VDR-KO) mice, there was an increase in visceral adipose tissue accompanied by an increase in hepatic lipid content and hepatic expression of genes related to fatty acid transport, synthesis, and oxidation [60]. VDR expression in the liver was studied in NAFLD mouse models and in patients with hepatosteatosis and NASH [61]. Induction of VDR expression was observed in NAFLD mouse models and in patients with hepatosteatosis, whereas VDR expression in the liver was decreased in NASH. Deletion of VDR in apoE^−/−^ mice fed a high-fat diet showed a protective effect on fatty liver, dyslipidemia, and insulin resistance. VDR in the liver was also associated with lipid metabolism. In the livers of apoE^−/−^VDR^−/−^ mice fed a high-fat diet, decreased expression of major lipogenic genes (CD36, DGAT2, and C/EBPα) and increased expression of genes involved in fatty acid utilization (PNPLA2/ATGL, LIPIN1, and PGC1α) were observed. Therefore, it was suggested that induction of hepatic VDR expression contributes to fat-associated hepatic steatosis by promoting hepatic lipogenesis and inhibiting lipid oxidation pathways [61]. 

The potential role of VDR in the development of NAFLD was also highlighted by Jahn et al. [62], who suggested a VDR-mediated metabolic cross-talk between gut and adipose tissue that could significantly affect systemic lipid homeostasis. Deletion of VDR in mouse intestine resulted in protection against diet-induced obesity, hepatosteatosis, and metabolic inflammation in liver and adipose tissue. These protective effects were possibly related to the observed lipoprotein lipase (LPL) in adipose tissue [62]. LPL is an enzyme that contributes to the hydrolysis and distribution of fatty acids in extrahepatic tissues and the uptake of lipoprotein particles from cells [63]. VDR^−/−^ mice also showed a decreased uptake of triglycerides from the circulation. These phenotypes were reversed in mice reexpressing an intestinal-specific human VDR transgene in the VDR deficiency model (VDR^−/−^hTg), indicating VDR activity in lipid homeostasis [62]. 

A link between intestinal VDR and the LPL enzyme was suggested by possible transcriptional effects of the VDR on the LPL inhibitor angiopoietin-like 4 (ANGPTL4) gene. ANGPTL4 gene expression was significantly increased in VDR^−/−^ mice compared with VDR^+/−^ mice [62]. The VDR-ANGPTL axis was suggested to promote the development of NAFLD. ANGPTL3 is a hepatokine regulated by VDR that inhibits LPL activity; experimentally induced inactivation of ANGPTL3 results in decreased hepatosteatosis [64]. Obese NAFLD patients were found to have a higher expression of VDR, ANGPTL3, and LPL in the liver compared to patients without NAFLD. ANGPTL3 correlated with the degree of steatosis and the expression of LPL, VDR, and enzymes related to vitamin D and cholesterol metabolism (CYP27A1 and CYP2R1). Higher hepatic ANGPTL3 expression resulted in higher plasma ANGPTL3 levels, which were positively associated with clinical/histological markers of NAFLD/NASH. Upregulation of hepatic VDR expression in NAFLD was the main modulator of the increase in hepatic ANGPTL3 expression, highlighting the significant role of VDR in ANGPTLs-mediated ectopic fat accumulation and the development of NAFLD in obese individuals [64]. ANGPTL8 expression was shown to increase upon VDR activation in hepatocytes [65]. Hepatic mRNA VDR levels and the expression of genes downstream the VDR-D pathway [cytochrome P450 (CYP) 2R1, CYP27A1, and CYP3A4] were also increased in patients with nonalcoholic fatty liver (NAFL) compared to healthy individuals [65]. Increased ANGPTL8 mRNA and protein levels and a positive correlation between ANGPTL8 mRNA and VDR mRNA and the degree of steatosis were found in NAFL patients [65]. The upregulation of both VDR and ANGPTL8 mRNA can be mediated by FFAs in human hepatocytes, and knockdown of the ANGPTL8 gene attenuated the FFA-induced TG accumulation in the liver. Therefore, activation of VDR was thought to promote hepatic TG accumulation by upregulating ANGPTL8 expression [65]. 

Although a negative role of VDR in NAFLD was previously described, there are also data reporting a protective effect of D-VDR signaling on NAFLD development. For example, a significant decrease in serum vitamin D levels was observed in NAFLD patients and mice fed a high-fat diet compared with healthy controls and mice fed chow, respectively [66]. Vitamin D supplementation improved high-fat diet-induced hepatic steatosis and insulin resistance in vivo [66]. In three NAFLD mouse models (high-fat diet-fed mice, methionine/choline-deficient diet-fed mice, and genetically obese ob/ob mice), upregulation of hepatic VDR expression was observed. Liver-specific deletion of VDR significantly exacerbated hepatic steatosis and insulin resistance and abrogated the protective effect of vitamin D on NAFLD [66]. VDR was shown to interact with hepatocyte nuclear factor 4 (HNF4), which regulates gene expression related to TG transport [66,67]. Overexpression of HNF4 ameliorated NAFLD and metabolic abnormalities in VDR-KO mice [66]. Therefore, vitamin D could prevent or improve NAFLD and metabolic abnormalities by activating the hepatic VDR-HNF4 interaction [66] (Table in Section 9, Figure 2).

### 7.2. Indirect Effects of VDR on the Development of NAFLD

There are several associated signaling pathways thought to mediate abnormal fatty acid influx and metabolism in the liver, and their roles were evaluated [68]. However, the elucidation of the mechanisms involved in the development of fatty liver is still ongoing. Insulin resistance (IR), proinflammatory stimuli, oxidative imbalance, alterations in the gut microbiota, vitamin D deficiency, and thyroid dysfunction are thought to contribute to lipid deposition in hepatocytes [69]. VDR may have an indirect effect on the development of NAFLD, as it is involved in some of these factors.

## 8. VDR and Insulin Resistance

Insulin resistance is thought to promote hepatic steatosis by increasing the influx of FFAs into the liver and de novo lipogenesis [70]. Imbalanced insulin substrate 1 (IRS1) and insulin substrate 2 (IRS2) signaling was shown to induce hepatic steatosis in vivo and was linked to VDR-D signaling [71]. Vitamin D-deficient rats showed increased expression of enzymes and transcription factors related to de novo lipogenesis and altered insulin receptor signaling. IRS1 signaling was increased, whereas IRS2 signaling was decreased under the influence of vitamin D deficiency. Treatment of vitamin D-deficient rats with a vitamin D analog (calcipotriol) or 8-methoxypsoralen (8-MOP), a vitamin D receptor agonist, decreased IRS1 signaling, resulting in decreased de novo lipogenesis. Administration of vitamin D and 8-MOP increased IRS2 expression, which was associated with nuclear suppression of forkhead box O1 (FoxO1) and decreased gluconeogenesis, which produces acetyl-CoA for de novo lipogenesis. Treatment of human hepatocyte cell lines with calcipotriol and 8-MOP also modulated insulin signaling. The inhibitory effect of calcipotriol and 8-MOP on fatty acid synthase and acetyl- CoA carboxylase 1 was abolished after VDR expression was silenced in vitro [71]. Activation of VDR by calcipotriol improved insulin sensitivity and reduced hepatic steatosis in mouse liver macrophages. Specifically, VDR activation in liver macrophages significantly increased the glucose infusion rate and decreased hepatic glucose production [72]. Deletion of VDR in mouse macrophages was associated with the induction of insulin resistance, as it led to accumulation of M2 macrophages in the liver, increased cytokine secretion, and hepatic glucose production. VDR KO macrophages showed increased mRNA expression of gluconeogenic enzymes (Pck1, G6pc) and secretion of TNFα IL-1β and IL-6 cytokines, which can induce hepatic gluconeogenesis [73]. VDR expression was also shown to determine the ability to secrete insulin in peripheral blood mononuclear cells (PBMCs) [74] (Figure 2).

## 9. VDR and Gut Microbiota

The VDR/D axis was shown to regulate the homeostasis of the gut microbiota by inducing Paneth cell defensins in the gut [75]. Gut dysbiosis is associated with impaired integrity of the intestinal epithelial barrier, leading to induction of systemic inflammation and translocation of bacteria to the liver, which can promote hepatic steatosis and insulin resistance [76,77,78]. Impairments in hepatic carbohydrate and lipid metabolism and an imbalance between pro- and anti-inflammatory factors in the liver are associated with gut dysbiosis [78]. The high expression of VDR in the ileum of the small intestine led to the hypothesis that vitamin D signaling may regulate the homeostasis of the gut microbiota [75]. The anti-microbial proteins, Paneth cell-specific α-defensins (α-defensin 5 (DEFA5), MMP7), are suppressed under the influence of high fat diet and vitamin D deficiency (HFD + VDD), resulting in impaired gut integrity, endotoxemia, systemic inflammation, and dysbiosis [75]. The presence of HFD + VDD and VDR deletion in mice was also associated with an increase in liver pathogens and a decrease in beneficial symbionts, highlighting the important role of VDR/D in gut microbiota-mediated pathogenesis of NAFLD [75]. VDR status was shown to modulate the composition and functions of the gut microbiota [79], and deletion of VDR in the gut can lead to dysbiosis [80]. Vitamin D may control the expression of anti-microbial peptides by binding to VDR, exert protective effects on the integrity of the intestinal epithelial barrier, and modulate immune responses against intestinal microbial pathogens, while metabolites of the gut microbiota may influence VDR expression [81]. The important role of VDR in maintaining a healthy gut microbiome was reported previously. Tissue-specific deletion of VDR in intestinal or myeloid cells in mice was associated with negative changes in microbial metabolites related to carbohydrate, protein, lipid, and bile acid metabolism [82] (Table 1).

## 10. The Role of Vitamin D-VDR Signaling in NAFLD Disease Progression

NAFLD comprises several distinct disease phenotypes. It begins with simple steatosis, called NAFL, and progresses to NASH under the influence of other pathogenic factors. NASH may progress to liver fibrosis, leading to cirrhosis and HCC. There are several reversible cycles between the NAFL and NASH stages before cirrhosis occurs. Around 10–20% of these cycles rapidly progress to fibrosis, and 80–90% and 10–20% of NASH patients show slow and rapid progression to cirrhosis and HCC, respectively [69].

## 11. Vitamin D-VDR and NASH

### 11.1. VDR and Lipotoxicity in NASH

The progression of NAFL to NASH is mediated by lipid-induced lipotoxicity in the liver, which in turn promotes the initiation of inflammatory responses, oxidative stress, and fibrosis [83]. Lipid overload in NAFL leads to the increased release of specific toxic compounds, including nonesterified fatty acids and ceramides, which further damage hepatocytes. The cellular damage triggered by toxic lipids is mediated by increased oxidative stress and mitochondrial dysfunction [84]. FFAs are normally metabolized in mitochondria by beta-oxidation. The increased FFA influx into mitochondria leads to mitochondrial uncoupling and production of reactive oxygen species (ROS) [83]. The positive role of VDR in mitochondrial function was highlighted in several human cell lines [85]. VDR silencing in vitro resulted in increased respiratory activity in mitochondria, leading to increased production of ROS. VDR was shown to control mitochondrial and nuclear transcription of genes (COX2, COX4, MT-ATP6, and ATP5B) involved in respiratory activity and ATP synthesis [85]. 

The abundance of saturated fatty acids in NASH can trigger inflammation and apoptosis of hepatocytes through the activation of mitochondrial signaling pathways and Jun N-terminal kinase (JNK), a stress mediator in the endoplasmic reticulum (ER) [86,87]. ER stress contributes to the development of NASH by triggering the unfolded protein response, a potent activator of apoptosis [87]. Activation of VDR in liver macrophages was shown to protect against ER stress in mice [88]. Notably, deletion of VDR in vivo was associated with sustained apoptosis and activation of the unfolded protein response under chemically induced ER stress. VDR deficiency resulted in increased infiltration of liver macrophages and expression of the proinflammatory cytokines IL-1β, IL-6, and TNF-α. Primary hepatocytes co-cultured with VDR-activated macrophages showed suppressed expression of genes involved in unfolded protein response, suggesting that VDR-mediated immune modulation of macrophages may contribute to the resolution of hepatic ER stress [88]. Vitamin D supplementation in transformed human mammary epithelial cells (MECs) showed protective effects against chemically induced ER stress. In vitro-induced ER stress resulted in decreased expression of VDR, which was reversed after vitamin D supplementation. Vitamin D activated the expression of VDR and inhibited the expression of genes related to the induction of ER stress [89] (Table in Section 13.2, Figure 2).

### 11.2. VDR and Immune Modulation in NASH

Chronic inflammation, a crucial pathogenic factor for NASH, may result from the prolonged death of hepatocytes, which promotes the induction of signaling pathways, including the tumor necrosis factor (TNF)-related apoptosis-inducing ligand receptor, Fas, and the TNF receptor, leading to the expression of various cytokines and chemokines [90]. Kupffer cells, together with other infiltrating immune cells, shape the proinflammatory milieu, leading to NASH. Uptake of FFAs by Kupffer cells causes them to adopt an inflammatory phenotype that secretes inflammatory cytokines, such as IL-6 and TNF-α [91]. The differentiation of Kupffer cells into the inflammatory M1 phenotype is determined by the interaction between Toll-like receptors (TLRs) and pathogen-associated molecular patterns (PAMPs) and damage-associated molecular patterns (DAMPs), which induces the expression of proinflammatory factors, including IL-1, IL-12, and CCL2 and CCL5 chemokines [90]. Immune cells, including T cells, B cells, dendritic cells (DCs), and liver macrophages, are able to synthesize 1,25(OH)_2_D_3_ and express VDR in response to their activation due to inflammation and liver injury [14,92,93].

Vitamin D may exert immunomodulatory effects on the liver by binding to VDR. Vitamin D supplementation resulted in the increased phagocytic activity of macrophages and secretion of anti-microbial peptides in vitro [94]. Activation of VDR in macrophages induced immunosuppression, resulting in under-expression of MHC II molecules that present antigens [94]. VDR may also mediate immunosuppression by maintaining mitochondrial function and preventing increased production of ROS, which triggers proinflammatory signaling (MAPK, STAT1, STAT6, and NF-κB) in macrophages [85,95]. VDR activation in liver macrophages was shown to downregulate the expression of NF-κB, an important modulator of inflammatory responses, leading to amelioration of liver inflammation [72]. Vitamin D-VDR was shown to prevent TLR-mediated induction of proinflammatory cytokines in liver macrophages by regulating the miR-155/SOCS1 negative feedback loop [96]. Vitamin D-VDR signaling was shown to inhibit T cell proliferation and production of the proinflammatory cytokines IFN-γ, IL-2, and IL-17 [14], and increase the activity of regulatory T cells (Tregs) and the production of the anti-inflammatory cytokines IL-4, IL-10, and TGF-β [93,97] (Table in Section 13.2, Figure 2).

## 12. Vitamin D-VDR and Fibrosis-Liver Cirrhosis

### 12.1. VDR and HSCs Activation

NASH is a chronic inflammatory disease of the liver that can lead to tissue damage. Immune cell infiltration during liver injury can activate HSCs and induce their differentiation from a quiescent phenotype to proliferative and contractile collagen-producing myofibroblasts [98]. Sustained inflammatory responses increase hepatocyte death and apoptosis. The release of DAMPs by dying hepatocytes sends danger signals to surrounding cells [99]. DAMPs are also released by apoptosis, and apoptotic bodies are phagocytosed by HSCs and Kupffer cells, triggering a pro-fibrogenic response. DNA from apoptotic hepatocytes was shown to induce hepatic HSC differentiation and collagen production [100]. Activation of VDR in HSCs was shown to inhibit fibrosis and liver inflammation [101]. In particular, p62/SQSTM1, a protein expressed by parenchymal liver cells, can negatively affect HSC activation by binding to VDR. Additionally, p62 was shown to directly interact with VDR and RXR and enhance their heterodimerization, leading to VDR/RXR-mediated induction of anti-inflammatory and anti-fibrotic gene expression [101]. In the presence of VDR agonists, deletion of p62 in HSCs does not prevent inflammation and fibrosis progression. Therefore, p62 may act as a negative regulator of liver fibrosis by stimulating VDR signaling in HSCs [101]. Vitamin D-VDR signaling also has an anti-proliferative and anti-fibrotic effect on HSCs by suppressing the expression of cyclin-D1 and collagen Iα1 [102]. The combined treatment of primary HSCs with 1,25(OH)_2_D_3_ and farnesylthiosalicylic acid, a Ras antagonist that inhibits liver fibrosis, was shown to have an anti-proliferative effect on HSCs. This anti-proliferative effect was found to be mediated via the Ras-GTP and p-ERK signal transduction pathway, leading to the suppression of cyclin D1 expression [103] (Table in Section 13.2).

### 12.2. VDR and MMPs/TIMPs

Specific metalloproteinases (MMPs) that degrade ECM components are increased in response to increased collagen production [104]. The increased MMPs in conjunction with the high collagen production lead to an overload of the ECM [105]. Physiologically, the activity of MMPs is controlled by the tissue inhibitors of metalloproteinases (TIMPs). With prolonged liver injury in NASH, the balance between MMPs and TIMPs is disturbed, leading to excessive deposition of ECM and fibrogenesis [106]. The addition of 1,25(OH)_2_D_3_ in cultured primary human uterine fibroid cells was shown to regulate the expression and activity of MMPs/TIMPs. Additionally, 1,25(OH)_2_D_3_ significantly induced the expression of VDR and TIMP-2 and decreased the protein levels of MMP-2 and MMP-9 in vitro, while the gelatinolytic activity of MMP-2 and MMP-9 was reduced in the presence of 1,25(OH)_2_D_3_ [107]. Vitamin D-VDR signaling was also shown to affect cardiac ECM metabolism in vivo by modulating the expression of MMPs/TIMPs. In VDR KO mice, increased fibrotic lesions, decreased expression of TIMP-1 and TIMP-3, and upregulation of MMP-2 and MMP-9 were observed [108] (Table in Section 13.2).

### 12.3. VDR and Fibrosis-Related Signal Transduction Pathways

Non-parenchymal cells, including Kupffer cells and other immune cells, myofibroblasts, and hepatic progenitor cells can produce fibrogenic cytokines and growth factors, leading to the activation of HSCs and recruitment of inflammatory cells [106]. There is a complex network of cytokine-induced signaling pathways that mediate profibrogenic cell interactions. Transforming growth factor beta (TGF-β), platelet-derived growth factor (PDGF), inflammasome (NLRP3)-caspase 1, and WNT/β-catenin signaling pathways are thought to be associated with HSC activation and fibrosis progression [109]. Sustained fibrogenesis leads to the formation of regenerative nodules with fibrous tissue and a collagenous scar encapsulating the injured liver parenchyma, a stage termed cirrhosis [110]. Additionally, 1,25(OH)_2_D_3_ prevented the progression of liver fibrosis in vivo by inhibiting the expression of PDGF and TGF-β collagen Iα1, metalloproteinase inhibitor-1, and alpha smooth muscle actin [111].

During liver fibrosis, binding of PDGF to the PDGFR receptor can activate proliferation of HSCs. PDGF-PDGFR signaling activates phosphorylation of intracellular protein kinases (PI3K, JAK1, and PLCγ) and induces Raf/MEK/ERK, JAK/STAT, and NF-κΒ signaling pathways that regulate gene expression associated with inflammation, fibrosis, and cell proliferation/apoptosis [112]. PDGF-A mRNA levels were upregulated in vitro after vitamin D treatment of a VDR-expressing clone of JEG-3 cells, suggesting that PDGF-A is a target gene of the vitamin D-VDR pathway [113]. The TGF-β1/SMAD pathway promotes the pro-fibrogenic response in HSCs. TGF-β1 is secreted in liver injury and binds to serine/threonine kinase receptors on HSCs to induce intracellular phosphorylation of its downstream effectors, SMAD2 and SMAD3, which form a complex with SMAD4. The SMAD complex migrates to the nucleus to recognize SMAD-binding elements (SBE) on the genome and regulate the expression of profibrotic genes [114]. TGF-β1 signaling can facilitate VDR binding to profibrotic SMAD3 genes by rearranging genome-wide VDR binding sites (VDR cistrome) in HSCs through chromatin remodeling. When vitamin D binds to VDR, VDR prevents SMAD3 from binding to profibrotic target genes, inhibiting fibrosis [115]. Vitamin D-VDR signaling was reported to attenuate TGF-β1-induced fibrosis, and vitamin D supplementation reduces VDR degradation in NAFLD liver [116]. 

Activation of the NLRP3 inflammasome in HSCs was associated with increased liver fibrogenesis in vivo [117]. Calcipotriol, a VDR agonist, was shown to ameliorate cholestatic liver injury and fibrosis through activation of yes-associated protein 1 (YAP1). Specifically, the binding of vitamin D to VDR induced YAP1 gene expression in the liver; the increased YAP1 activity resulted in inhibition of the NLRP3 inflammasome and subsequent liver injury and fibrogenesis [117]. It was also reported that VDR is a negative regulator of NLRP3 activation in vivo. VDR can bind to NLRP3 and prevent the process of deubiquitination (NLRP3 activation) mediated by the interaction between NLRP3 and BRCC3 [118]. WNT/β-catenin signaling contributes to normal liver development and regeneration, and key-molecules (LRP6, Wnt1, Wnt3a, β-catenin, GSK-3β, and APC) regulating WNT/β-catenin signaling are disrupted in the progression of NAFLD to NASH [119]. Once β-catenin enters the nucleus, it interacts with TCF-LEF co-transcription factors to stimulate the transcription of genes related to cell proliferation and fibrosis (cyclin-D1, MYC, MMP7, and fibronectin) [120]. The WNT/β-catenin pathway was reported to promote fibrogenesis along with TGF-β signaling [121]. VDR agonists, including vitamin D_3_ and lithocholic acid (LCA), were associated with downregulation of the transactivating activity of β-catenin/TCF, suppressing the expression of downstream genes [122]. In addition, VDR KO resulted in a vitamin D-mediated blockade of β-catenin transport from the nucleus to the cell membrane [122]. Decreased VDR signaling in A549 cells was associated with increased activation of the Wnt/β-catenin pathway, induction of an epithelial–mesenchymal transition, and myofibroblast differentiation [123] (Table in Section 13.2, Figure 2).

## 13. VDR and HCC

NASH, advanced liver fibrosis, and cirrhosis are associated with an increased risk for developing HCC [124,125]. A dynamic network of different cell types, including cancer-associated fibroblasts (CAFs), endothelial cells, B and T cells, neutrophils, and tumor-associated macrophages (TAMs), was demonstrated in the tumor microenvironment in HCC [126].

### 13.1. VDR and CAFs

CAFs, likely derived from HSCs, can differentiate into ECM-producing myofibroblasts and interact with cancer cells, affecting tumor growth and invasion. A potential pathogenic activity of CAFs in HCC was highlighted because CAFs can influence tumorigenesis by altering ECM stiffness and secreting cytokines and other factors that contribute to tumor growth, angiogenesis, and epithelial-to-mesenchymal transition [126]. A role of VDR in the activity of CAFs in tumor progression was highlighted. VDR signaling was shown to abrogate the pro-tumor effects of CAFs in pancreatic cancer by suppressing the secretion of exosomal miR-10a-5p [127]. 

A beneficial role of vitamin D and VDR, related to their activity on stromal fibroblasts, was reported in patients with colorectal cancer (CRC), in whom increased VDR expression in stromal tumor fibroblasts was associated with better survival [128]. It was suggested that vitamin D-VDR signaling regulates stromal fibroblasts by inhibiting pro-tumoral activation of CAFs and determines a vitamin D-associated gene signature (CD82 and S100A4) in CAFs that correlates with stromal VDR expression and better clinical outcome in CRC [128]. CAFs and normal mammary-associated fibroblasts (NAFs) express VDR and show an altered transcriptional profile after vitamin D treatment [129]. In CAFs, vitamin D-VDR-mediated downregulation of genes related to the proliferation (NRG1, WNT5A, and PDGFC) and upregulation of genes involved in immune modulation (NFKBIA, TREM-1) were observed [129]. Induction of genes regulating apoptosis, detoxification, anti-bacterial defense, and protection against oxidative stress was reported in NAFs. Therefore, the vitamin D-VDR pathway may limit carcinogenesis by modulating the gene expression profile in CAFs/NAFs [129]. The vitamin D-VDR pathway could enhance the anti-tumor effect of chemotherapy in gastric cancer (GC) by inhibiting the tumor-supporting activity of CAFs [130]. Specifically, activation of VDR in vitamin D-treated GC cells prevented CAF-derived IL-8-mediated chemotherapy resistance by blocking PI3K/Akt signaling [130]. 

CAFs can express growth factors (FGF, PDGF, and VEGF) and their respective receptors that promote angiogenesis and tumor growth [131,132,133]. The role of hepatic fibroblast growth factor (FGF21) was described in the NASH transition to HCC in vivo [134]. FGF21 KO mice showed upregulation of hepatocyte-derived IL-17A. It was suggested that FGF21 exerts anti-inflammatory and anti-carcinogenic effects by inhibiting hepatocyte TLR4-IL-17A signaling in the NASH-HCC mouse model, which may prevent the development of HCC. IL-17A expression triggered by TLR4 signaling in hepatocytes was attenuated after FGF21 restoration, and anti-IL17 treatment reduced HCC tumor size [134]. It was reported that the Th17-IL-17 axis mediates NASH progression to HCC [135]. Hepatic deletion of VDR in vivo was associated with decreased expression of FGF21, suggesting that FGF21 is a target of VDR signaling [61,136]. PDGF signaling may promote angiogenesis by inducing transcription and secretion of VEGF [137]. The PDGF-A gene was proposed as a VDR target because PDGF-A mRNA levels were upregulated in vitro after the addition of vitamin D to a VDR-expressing clone of JEG-3 cells [113]. Vitamin D-VDR signaling was shown to affect angiogenesis in vivo by modulating VEGF expression and signaling [138] (Table 2).

### 13.2. VDR and CAFs-Immune Cell Crosstalk

T lymphocytes, including CD8+ cytotoxic T lymphocytes (CTLs) and CD4+ Th1 cells, natural killer (NK) cells, and DCs are major players in tumor-associated immune responses [139]. Tumor development can be promoted by the cancer cell-mediated recruitment of immunosuppressive cells, including Tregs and myeloid-derived suppressor cells (MDSCs), which inhibit the activity of effector T cells [140,141]. CAFs can disrupt immune surveillance, promoting tumor immune escape and the development of HCC [142]. The vitamin D-VDR pathway may exert anti-tumor effects in the development of HCC by modulating the anti-tumor immune responses and restoring the disruption of immune homeostasis caused by CAFs. CAFs are thought to reduce T cell activation by inhibiting the activity of DCs and promoting the differentiation of T cells into an immunosuppressive phenotype (Tregs) [142]. CAFs can also impede anti-tumor toxicity by inhibiting the cytotoxic activity of CTL and NK cells on tumor cells, leading to tumor immune escape and T cell inhibition by recruiting MDSCs and inducing their differentiation into immunosuppressive phenotypes [142]. The vitamin D-VDR pathway was reported to restore exhausted CTLs and promote anti-tumor immunity in patients with lung adenocarcinoma [143]. Binding of vitamin D to VDR resulted in decreased expression of immune checkpoint inhibitors (PD-1, TIGIT, and Tim-3) and increased expression of the co-stimulatory molecule CD28 on CD8+ T cells, which increased their cytokine production and anti-tumor activity [143]. VDR-mediated Ca^2+^ influx resulted in increased expression of Th1 cytokines in vitamin D-treated CD8+ T cells or Vγ9Vδ2+ T cells through activation of the T cell receptor (TCR) [143]. The beneficial effect of vitamin D-VDR signaling on anti-tumor immunity was highlighted in head and neck squamous cell carcinoma (HNSCC). Vitamin D supplementation in HNSCC patients was associated with a significant increase in NK cytotoxic activity [144]. VDR signaling is thought to promote anti-tumor immunity by inhibiting the Wnt-beta catenin pathway [145]. Melanoma cells with active VDR signaling and a suppressed Wnt-beta catenin pathway show increased tumor antigen release, antigen presentation, CD4+ T cell activation and priming, and increased CTL and NK infiltration and tumor cell killing [145]. 

CAFs can promote tumor cell tolerance by regulating M1-M2 polarization of macrophages and maintain tumorigenic chronic inflammation by producing factors (CXCLs, CCLs, IL-6, SDF-1, and Chi3L1) that recruit immune cells [142]. Vitamin D-VDR signaling can alleviate the chronic inflammation induced by CAFs and thus prevent the development of HCC. VDR was shown to interact with the inhibitor of the NF-κB subunit beta (IKBKB) to block the activation of NF-κB, a transcription factor involved in the expression of inflammatory cytokines and the differentiation of inflammatory cells [146,147]. Vitamin D-VDR activity mediated the upregulation of cyclin-dependent kinase inhibitor (CKI) p27^kip1^ gene expression and the reduction in the release of proinflammatory cytokines IL-6 and TNF-α from macrophages and lymphocytes [148]. The vitamin D-VDR pathway can modulate innate immunity by enhancing the expression of anti-microbial peptides and T cell responses, suppressing inflammatory Th1 and Th17 cells, and inducing tolerogenic Treg responses [149]. The role of the vitamin D-VDR complex in regulating the immune response is related to its involvement in TCR signaling and T cell activation. Antigen-presenting cells express VDR and activated T cells increase their VDR expression [150]. Vitamin D-VDR signaling can suppress T cell activation and proliferation [151], attenuate Th1- and Th17-driven inflammatory responses [152], and enhance the anti-inflammatory activity of Th2 cells [153]. Vitamin D can induce the differentiation of DCs into a tolerogenic phenotype that promotes the differentiation of effector T cells into immunosuppressive Tregs [154] (Table 2, Figure 2).

**Table 2:** Effects of vitamin D-VDR signaling on NAFLD disease progression.

VDR Mediated-Mechanism	Effects on NAFLD Disease Progression	Refs.
VDR and lipotoxicity in NASH		
VDR → control of COX2, COX4, MT-ATP6, ATP5B gene expression → mitochondrial function → regulation of ROS production VDR activation in macrophages → suppressed expression of genes, implicated in unfolded protein response → protection against ER stress	Regulation of toxic lipid-induced cell injury → NAFL progression to NASH Prevention of ER stress-induced NASH development	[83,84,85,86,87,88,89]
VDR and immune modulation in NASH		
VDR → ↑phagocytic activity of macrophages, anti-microbial peptides VDR → ↓MHC II antigen presentation VDR → ↓ROS → induce proinflammatory signaling (MAPK, STAT1, STAT6 and NF-κB) VDR activation in hepatic macrophages → regulation of NF-κB gene expression VDR → regulation of miR-155/SOCS1 → ↓TLR-mediated induction of proinflammatory cytokines VDR → ↓T cell proliferation and proinflammatory cytokines (IFN-γ, IL-2, IL-17) and ↑activity of Tregs and anti-inflammatory cytokines (IL-4, IL-10, TGF-β)	Modulation of inflammatory responses → improved chronic liver inflammation → ↓NASH development	[14,72,85,90,91,92,93,94,95,96,97]
VDR and fibrosis-liver cirrhosis		
VDR interacts with p62/SQSTM1 → control of HSC activation VDR → ↑Ras-GTP and p-ERK signal transduction pathway → ↓cyclin-D1 and collagen Iα1 gene expression → anti-proliferative and anti-fibrotic effects on HSCs	Reduced HSC proliferation and activity → ↓pro-fibrogenic response → ↓fibrosis development	[98,100,101,102,103]
VDR activation → modulation of MMPs/TIMPs expression → regulation of ECM metabolism	Prevention of disrupted MMPs/TIMPs balance → ↓ECM deposition → ↓fibrogenesis	[105,106,107,108]
VDR → regulation of PDGF expression → regulation of HSCs proliferation and proinflammatory/profibrotic activity of Raf/MEK/ERK, JAK/STAT, NF-κΒ signaling pathways VDR → ↓SMAD3 binding to profibrotic target genes → regulation of TGF-β1/SMAD pathway	↓pro-fibrogenic response in HSCs → ↓liver fibrosis progression	[111,112,113,114,115,116]
Calcipotriol → ↑YAP1 expression and activity → ↓NLRP3 inflammasome VDR binds to NLRP3 → ↓deubiquitination → ↓NLRP3 activation	↓liver injury and fibrogenesis	[117,118]
VDR agonists → ↓transactivational activity of β-catenin/TCF → ↓cyclin-D1, MYC, MMP7, fibronectin gene expression VDR → ↓WNT/β-catenin pathway interaction with TGF-β signaling	↓epithelial-mesenchymal transition and myofibroblast differentiation → ↓fibrogenesis	[119,120,121,122,123]
VDR and hepatocellular carcinoma (HCC)		
VDR → ↓exosomal miR-10a-5p → ↓protumoral effects of CAFs VDR → ↓protumoral activation of CAFs and determination of gene signature in CAFs, associated with VDR expression →regulation of stromal fibroblasts VDR → ↓NRG1, WNT5A, PDGFC gene expression (CAFs proliferation), ↑ NFKBIA, TREM-1 gene expression (immune modulation) and ↑gene expression (anti-apoptosis, detoxification, anti-bacterial defense and protection against oxidative stress) in NAFs VDR → ↓PI3K/Akt signaling → ↓CAF-derived IL-8-mediated chemotherapy resistance VDR → regulation of FGF, PDGF, VEGF gene expression in CAFs	↓tumor growth and angiogenesis → ↓HCC progression	[113,126,127,128,129,130,131,132,133]
VDR → restoration of exhausted CTLs phenotype, ↓expression of immune checkpoint inhibitors (PD-1, TIGIT, Tim-3) and ↑co-stimulatory molecule CD28 on CD8+ T cells → ↑cytokine production/anti-tumor immunity VDR-mediated Ca^2+^ influx → TCR activation → ↑Th1 cytokines D-VDR signaling → ↑NK cytotoxic activity VDR → ↓Wnt-beta catenin signaling pathway → ↑tumor antigen release, antigen presentation, CD4+ T cell activation and priming, CTL and NK infiltration and tumor cell killing	↑immune cell activation → ↑anti-tumor toxicity → ↓HCC progression	[142,143,144,145]
VDR interacts with IKBKB → ↓NF-κΒ activation → ↓proinflammatory cytokines VDR → ↑(CKI) p27kip1, ↓proinflammatory cytokines IL-6 and TNF-α from macrophages and lymphocytes VDR → ↑anti-microbial peptides, ↓inflammatory Th1 and Th17, ↑tolerogenic Treg responses VDR → ↓activation and proliferation of T cells, ↓Th1 and Th17- driven inflammatory responses, ↑anti-inflammatory Th2 cell activity D-VDR signaling → ↑DCs differentiation into a tolerogenic phenotype → ↑T effector cell differentiation into immunosuppressive Tregs ↑: increase, ↓: decrease	↓CAFs-mediated tumor immune tolerance → ↓HCC progression	[142,146,147,148,149,150,151,152,153,154]

## 14. Conclusions and Future Directions

The vitamin D-VDR axis is significantly associated with the development and progression of NAFLD. VDR gene polymorphisms were associated with the development and severity of NAFLD and may play an important role in the progression of NAFLD to NASH, fibrosis, and HCC. Future large-scale epidemiological studies examining the distribution and clinical outcomes of specific VDR gene polymorphisms in different NAFLD phenotypes will provide useful evidence to the research field of pharmacogenomics and personalized medicine. Conflicting data exist on the role of VDR in the development of NAFLD, as deletion of VDR in the liver showed both protective and detrimental effects on hepatic steatosis, lipid metabolism, and insulin resistance. Clinical maintenance of adequate vitamin D levels in adults was reported to be beneficial for both prevention and cure of NAFLD [155]. Elevated vitamin D levels were also associated with a lower risk of all-cause and cardiovascular death in NAFLD patients [156]. Therefore, the protective effects of vitamin D-VDR signaling on NAFLD should be further investigated in experimental and clinical studies. The vitamin D-VDR signaling pathway may control the development of NASH and HCC by modulating immune responses and ameliorating lipotoxicity. It may also inhibit fibrogenesis by controlling ECM metabolism, HSC activity, and profibrotic gene expression. The development of VDR agonists that exert anti-fibrotic and anti-tumor functions in the liver is ongoing and may represent a promising strategy for NAFLD disease progression. Calcipotriol, a novel VDR agonist, was shown to reduce liver fibrosis in vivo by inhibiting the activation of HSCs and the deposition of ECM [157]. Seocalcitol, a vitamin D analog, was tested for its anti-tumor effect in patients with HCC in a phase II study, where it showed a reduction in tumor size [158]. Elucidation of the VDR-related genetic and molecular background of NAFLD pathophysiology may lead to new therapeutic approaches targeting NAFLD via vitamin D-VDR signaling. 

## Figures and Tables

**Table 1:** Direct and indirect effects of VDR in NAFLD development.

VDR Mediated-Mechanism	Effects on NAFLD Development	Refs.
Direct effects of VDR		
Regulation of hepatic lipid accumulation VDR deletion → ↑hepatic expression of genes, related to fatty acid transport, synthesis and fatty acid oxidation	↑hepatic lipid content ↑visceral adipose tissue	[60]
↑VDR expression and gene expression downstream the VDR-D pathway [cytochrome P450 (CYP) 2R1, CYP27A1, and CYP3A4] in NAFLD → regulation of lipid metabolism VDR deletion → ↓lipogenic genes (CD36, DGAT2, C/EBPα), ↑genes of fatty acid utilization (PNPLA2/ATGL, LIPIN1 and PGC1α)	↑hepatic lipogenesis ↓lipid oxidation pathways high-fat-associated liver steatosis	[61,65]
VDR deletion → ↑ANGPTL4 expression → ↓LPL expression ↑hepatic VDR expression → ↑ANGPTL3 expression → ↓LPL activity Activation of VDR → ↑ANGPTL8 expression → ↑TG accumulation	Regulation of TG uptake from circulation, ↑hepatic TG accumulation VDR-ANGPTs-mediated ectopic fat accumulation Hepatosteatosis grade ANGPTL3 related to clinical/histological markers of NAFLD	[62,63,64,65]
VDR interacts with HNF4 → regulation of gene expression, related to TG transport	ameliorated high fat diet-induced hepatic steatosis and insulin resistance	[66,67]
Indirect effects of VDR		
Association between D-VDR signaling and imbalance of IRS1 and IRS2	↑insulin resistance → ↑FFAs influx and hepatic steatosis	[71]
Calcipotriol-induced VDR activation in hepatic macrophages → ↑glucose infusion rate and ↓hepatic glucose production	improved insulin sensitivity and reduced hepatic steatosis	[72]
VDR deletion → ↑hepatic glucose production, ↑mRNA expression of gluconeogenic enzymes (Pck1, G6pc) and secretion of TNFα IL-1β, and IL-6 cytokines → ↑hepatic gluconeogenesis VDR expression → determine insulin secretory capacity in PBMCs	↑insulin resistance → ↑hepatic steatosis	[71,73,74]
VDR-mediated metabolic cross-talk between gut and adipose tissue → systemic lipid homeostasis VDR/D axis → Paneth cell defensins → regulation of gut microbiota homeostasis → regulation of systemic inflammation and hepatic bacterial translocation VDR status → composition and functions of gut microbiota VDR deletion → gut dysbiosis and negative alterations in carbohydrate, protein, lipid, and bile acid metabolism	Regulation of gut dysbiosis-associated hepatic steatosis and insulin resistance	[62,76,77,78,79,80,81,82]
	↑: increase, ↓: decrease	

## Data Availability

No new data were created.

## References

[1] Younossi Z.M., Koenig A.B., Abdelatif D., Fazel Y., Henry L., Wymer M. (2016). Global epidemiology of nonalcoholic fatty liver disease-Meta-analytic assessment of prevalence, incidence, and outcomes. Hepatology.

[2] Estes C., Razavi H., Loomba R., Younossi Z., Sanyal A.J. (2018). Modeling the epidemic of nonalcoholic fatty liver disease demonstrates an exponential increase in burden of disease. Hepatology.

[3] Powell E.E., Wong V.W.S., Rinella M. (2021). Non-alcoholic fatty liver disease. Lancet.

[4] Mundi M.S., Velapati S., Patel J., Kellogg T.A., Abu Dayyeh B.K., Hurt R.T. (2020). Evolution of NAFLD and Its Management. Nutr. Clin. Pract..

[5] Kawagoe F., Mendoza A., Hayata Y., Asano L., Kotake K., Mototani S., Kawamura S., Kurosaki S., Akagi Y., Takemoto Y. (2021). Discovery of a Vitamin D Receptor-Silent Vitamin D Derivative That Impairs Sterol Regulatory Element-Binding Protein In Vivo. J. Med. Chem..

[6] Cimini F.A., Barchetta I., Carotti S., Morini S., Cavallo M.G. (2019). Overview of studies of the vitamin D/vitamin D receptor system in the development of non-alcoholic fatty liver disease. World J. Gastrointest. Pathophysiol..

[7] Barchetta I., Cimini F.A., Cavallo M.G. (2020). Vitamin D and Metabolic Dysfunction-Associated Fatty Liver Disease (MAFLD): An Update. Nutrients.

[8] Arai T., Atsukawa M., Tsubota A., Koeda M., Yoshida Y., Okubo T., Nakagawa A., Itokawa N., Kondo C., Nakatsuka K. (2019). Association of vitamin D levels and vitamin D-related gene polymorphisms with liver fibrosis in patients with biopsy-proven nonalcoholic fatty liver disease. Dig. Liver Dis..

[9] Yaghooti H., Ghanavati F., Seyedian S.S., Cheraghian B., Mohammadtaghvaei N. (2021). The efficacy of calcitriol treatment in non-alcoholic fatty liver patients with different genotypes of vitamin D receptor FokI polymorphism. BMC Pharmacol. Toxicol..

[10] Khan R.J., Riestra P., Gebreab S.Y., Wilson J.G., Gaye A., Xu R., Sharon D.K. (2016). Vitamin D Receptor Gene Polymorphisms Are Associated with Abdominal Visceral Adipose Tissue Volume and Serum Adipokine Concentrations but Not with Body Mass Index or Waist Circumference in African Americans: The Jackson Heart Study123. J. Nutr..

[11] Triantos C., Aggeletopoulou I., Kalafateli M., Spantidea P.I., Vourli G., Diamantopoulou G., Tapratzi D., Michalaki M., Manolakopoulos S., Gogos C. (2018). Prognostic significance of vitamin D receptor (VDR) gene polymorphisms in liver cirrhosis. Sci. Rep..

[12] Mosaad H., Emam E.A., Hamed E.F., El Demerdash E.A., Hussein S. (2020). Vitamin D receptor gene polymorphism and hepatocellular carcinoma in chronic hepatitis C patients. Egypt. Liver J..

[13] Nurminen V., Seuter S., Carlberg C. (2019). Primary Vitamin D Target Genes of Human Monocytes. Front. Physiol..

[14] Kongsbak M., Levring T.B., Geisler C., Rode von Essen M. (2013). The Vitamin D Receptor and T Cell Function. Front. Immunol..

[15] Arai H., Miyamoto K.I., Yoshida M., Yamamoto H., Taketani Y., Morita K., Kubota M., Yoshida S., Ikeda M., Watabe F. (2001). The polymorphism in the caudal-related homeodomain protein Cdx-2 binding element in the human vitamin D receptor gene. J. Bone Miner. Res..

[16] Fang Y., van Meurs J.B.J., d’Alesio A., Jhamai M., Zhao H., Rivadeneira F., Hofman A., van Leeuwen J.P.T., Jehan F., Pols H.A.P. (2005). Promoter and 3’-untranslated-region haplotypes in the vitamin d receptor gene predispose to osteoporotic fracture: The rotterdam study. Am. J. Hum. Genet..

[17] Arai H., Miyamoto K., Taketani Y., Yamamoto H., Iemori Y., Morita K., Tonai T., Nishisho T., Mori S., Takeda E. (1997). A vitamin D receptor gene polymorphism in the translation initiation codon: Effect on protein activity and relation to bone mineral density in Japanese women. J. Bone Miner. Res..

[18] Saccone D., Asani F., Bornman L. (2015). Regulation of the vitamin D receptor gene by environment, genetics and epigenetics. Gene.

[19] Morrison N.A., Qi J.C., Tokita A., Kelly P.J., Crofts L., Nguyen T.V., Sambrook P.N., Eisman J.A. (1994). Prediction of bone density from vitamin D receptor alleles. Nature.

[20] Ye W.Z., Reis A.F., Velho G. (2000). Identification of a novel Tru9 I polymorphism in the human vitamin D receptor gene. J. Hum. Genet..

[21] Selvaraj P., Prabhu Anand S., Harishankar M., Alagarasu K. (2009). Plasma 1,25 dihydroxy vitamin D3 level and expression of vitamin d receptor and cathelicidin in pulmonary tuberculosis. J. Clin. Immunol..

[22] Andraos C., Koorsen G., Knight J.C., Bornman L. (2011). Vitamin D receptor gene methylation is associated with ethnicity, tuberculosis, and TaqI polymorphism. Hum. Immunol..

[23] Uitterlinden A.G., Fang Y., Van Meurs J.B.J., Pols H.A.P., Van Leeuwen J.P.T.M. (2004). Genetics and biology of vitamin D receptor polymorphisms. Gene.

[24] Angulo P. (2007). GI epidemiology: Nonalcoholic fatty liver disease. Aliment. Pharmacol. Ther..

[25] Jaroenlapnopparat A., Suppakitjanusant P., Ponvilawan B., Charoenngam N. (2022). Vitamin D-Related Genetic Variations and Nonalcoholic Fatty Liver Disease: A Systematic Review. Int. J. Mol. Sci..

[26] Almeda-Valdés P., Cuevas-Ramos D., Aguilar-Salinas C.A. (2009). Metabolic syndrome and non-alcoholic fatty liver disease. Ann. Hepatol..

[27] Schuch N.J., Garcia V.C., Gouvea Ferreiro Vivolo S.R., Martini L.A. (2013). Relationship between Vitamin D Receptor gene polymorphisms and the components of metabolic syndrome. Nutr. J..

[28] Fabbrini E., Sullivan S., Klein S. (2010). Obesity and Nonalcoholic Fatty Liver Disease: Biochemical, Metabolic and Clinical Implications. Hepatology.

[29] Al-Daghri N.M., Guerini F.R., Al-Attas O.S., Alokail M.S., Alkharfy K.M., Draz H.M., Agliardi C., Costa A.S., Saulle I., Mohammed A.K. (2014). Vitamin D receptor gene polymorphisms are associated with obesity and inflammosome activity. PLoS ONE.

[30] Chiu K.W., Goto S., Nakano T., Hu T.H., Chen D.W., Huang K.T., Hsu L.W., Chen C.L. (2018). Genetic polymorphisms of the hepatic pathways of fatty liver disease after living donor liver transplantation. Liver Int..

[31] Petta S., Cammà C., Scazzone C., Tripodo C., Di Marco V., Bono A., Cabibi D., Licata G., Porcasi R., Marchesini G. (2010). Low vitamin D serum level is related to severe fibrosis and low responsiveness to interferon-based therapy in genotype 1 chronic hepatitis C. Hepatology.

[32] Jablonski K.L., Jovanovich A., Holmen J., Targher G., McFann K., Kendrick J., Chonchol M. (2013). Low 25-hydroxyvitamin D level is independently associated with non-alcoholic fatty liver disease. Nutr. Metab. Cardiovasc. Dis..

[33] Kneeman J.M., Misdraji J., Corey K.E. (2012). Secondary causes of nonalcoholic fatty liver disease. Ther. Adv. Gastroenterol..

[34] Vernon G., Baranova A., Younossi Z.M. (2011). Systematic review: The epidemiology and natural history of non-alcoholic fatty liver disease and non-alcoholic steatohepatitis in adults. Aliment. Pharmacol. Ther..

[35] Farrell G.C., Larter C.Z. (2006). Nonalcoholic fatty liver disease: From steatosis to cirrhosis. Hepatology.

[36] Ascha M.S., Hanouneh I.A., Lopez R., Tamimi T.A.R., Feldstein A.F., Zein N.N. (2010). The incidence and risk factors of hepatocellular carcinoma in patients with nonalcoholic steatohepatitis. Hepatology.

[37] Falleti E., Bitetto D., Fabris C., Cussigh A., Fontanini E., Fornasiere E., Fumolo E., Bignulin S., Cmet S., Minisini R. (2010). Vitamin D receptor gene polymorphisms and hepatocellular carcinoma in alcoholic cirrhosis. World J. Gastroenterol..

[38] Baur K., Mertens J.C., Schmitt J., Iwata R., Stieger B., Eloranta J.J., Frei P., Stickel F., Dill M.T., Seifert B. (2012). Combined effect of 25-OH vitamin D plasma levels and genetic vitamin D receptor (NR 1I1) variants on fibrosis progression rate in HCV patients. Liver Int..

[39] Hung C.H., Chiu Y.C., Hu T.H., Chen C.H., Lu S.N., Huang C.M., Wang J.H., Lee C.M. (2014). Significance of vitamin d receptor gene polymorphisms for risk of hepatocellular carcinoma in chronic hepatitis C. Transl. Oncol..

[40] Pontoriero A.C., Trinks J., Hulaniuk M.L., Caputo M., Fortuny L., Pratx L.B., Frias A., Torres O., Nunez F., Gadano A. (2015). Influence of ethnicity on the distribution of genetic polymorphisms associated with risk of chronic liver disease in South American populations. BMC Genet..

[41] Gibson P.S., Quaglia A., Dhawan A., Wu H., Lanham-New S., Hart K.H., Fitzpatrick E., Moore J.B. (2018). Vitamin D status and associated genetic polymorphisms in a cohort of UK children with non-alcoholic fatty liver disease. Pediatr. Obes..

[42] Heyens L.J.M., Busschots D., Koek G.H., Robaeys G., Francque S. (2021). Liver Fibrosis in Non-alcoholic Fatty Liver Disease: From Liver Biopsy to Non-invasive Biomarkers in Diagnosis and Treatment. Front. Med..

[43] Thanapirom K., Suksawatamnuay S., Sukeepaisarnjaroen W., Tangkijvanich P., Thaimai P., Wasitthankasem R., Poovorawan Y., Komolmit P. (2019). Genetic associations of vitamin D receptor polymorphisms with advanced liver fibrosis and response to pegylated interferon-based therapy in chronic hepatitis C. PeerJ.

[44] Gisbert-Ferrándiz L., Cosin-Roger J., Hernández C., Macias-Ceja D.C., Ortiz-Masiá D., Salvador P., Wildenberg M.E., Esplugues J.V., Alos R., Navarro F. (2020). The vitamin D receptor Taq I polymorphism is associated with reduced VDR and increased PDIA3 protein levels in human intestinal fibroblasts. J. Steroid Biochem. Mol. Biol..

[45] Li Y.J., Tang Y.W., Shi Y.Q., Han S., Wang J.B., Zhou X.M., Chen Y., Wu Z.D., Han Z.Y., Han Y. (2014). Polymorphisms in the vitamin D receptor gene and risk of primary biliary cirrhosis: A meta-analysis. J. Gastroenterol. Hepatol..

[46] Fang F., Wang J., Pan J., Su G.H., Xu L.X., Li G. (2015). Relationship between vitamin D (1,25-dihydroxyvitamin D3) receptor gene polymorphisms and primary biliary cirrhosis risk: A meta-analysis. Genet. Mol. Res..

[47] Luis C.B., Adams L.A. (2016). The Natural Course of Non-Alcoholic Fatty Liver Disease. Int. J. Mol. Sci..

[48] Quan Y., Yang J., Qin T., Hu Y. (2019). Associations between twelve common gene polymorphisms and susceptibility to hepatocellular carcinoma: Evidence from a meta-analysis. World J. Surg. Oncol..

[49] Barooah P., Saikia S., Bharadwaj R., Sarmah P., Bhattacharyya M., Goswami B., Medhi S. (2019). Role of VDR, GC, and CYP2R1 Polymorphisms in the Development of Hepatocellular Carcinoma in Hepatitis C Virus-Infected Patients. Genet. Test. Mol. Biomark..

[50] Stokes C.S., Volmer D.A., Grünhage F., Lammert F. (2013). Vitamin D in chronic liver disease. Liver Int..

[51] Gascon-Barré M., Demers C., Mirshahi A., Néron S., Zalzal S., Nanci A. (2003). The normal liver harbors the vitamin D nuclear receptor in nonparenchymal and biliary epithelial cells. Hepatology.

[52] Zúñiga S., Firrincieli D., Housset C., Chignard N. (2011). Vitamin D and the vitamin D receptor in liver pathophysiology. Clin. Res. Hepatol. Gastroenterol..

[53] Bikle D.D., Feingold K.R., Anawalt B., Blackman M.R., Boyce A., Chrousos G., Corpas E., de Herder W.W., Dhatariya K., Dungan K. Vitamin D: Production, Metabolism and Mechanisms of Action. http://www.ncbi.nlm.nih.gov/books/NBK278935/.

[54] Scott M.J. (2019). The upside-downside nature of Vitamin D signaling in liver. J. Leukoc. Biol..

[55] Bagur R., Hajnóczky G. (2017). Intracellular Ca2+ sensing: Role in calcium homeostasis and signaling. Mol. Cell.

[56] Mota M., Banini B.A., Cazanave S.C., Sanyal A.J. (2016). Molecular mechanisms of lipotoxicity and glucotoxicity in nonalcoholic fatty liver disease. Metabolism.

[57] Geng Y., Faber K.N., de Meijer V.E., Blokzijl H., Moshage H. (2021). How does hepatic lipid accumulation lead to lipotoxicity in non-alcoholic fatty liver disease?. Hepatol. Int..

[58] Bugianesi E., McCullough A.J., Marchesini G. (2005). Insulin resistance: A metabolic pathway to chronic liver disease. Hepatology.

[59] Donnelly K.L., Smith C.I., Schwarzenberg S.J., Jessurun J., Boldt M.D., Parks E.J. (2005). Sources of fatty acids stored in liver and secreted via lipoproteins in patients with nonalcoholic fatty liver disease. J. Clin. Invest..

[60] Tao T., Kobelski M.M., Saini V., Demay M.B. (2022). Adipose-specific VDR Deletion Leads to Hepatic Steatosis in Female Mice Fed a Low-Fat Diet. Endocrinology.

[61] Bozic M., Guzman C., Benet M., Sanchez-Campos S., Garcia-Monzon C., Gari E., Gatius S., Valdivielso J.M., Jover R. (2016). Hepatocyte vitamin D receptor regulates lipid metabolism and mediates experimental diet-induced steatosis. J. Hepatol..

[62] Jahn D., Dorbath D., Schilling A.K., Gildein L., Meier C., Vuille-Dit-Bille R.N., Schmitt J., Kraus D., Fleet J.C., Hermanns H.M. (2019). Intestinal vitamin D receptor modulates lipid metabolism, adipose tissue inflammation and liver steatosis in obese mice. Biochim. Biophys. Acta Mol. Basis Dis..

[63] Zechner R., Strauss J., Frank S., Wagner E., Hofmann W., Kratky D., Hiden M., Levak-Frank S. (2000). The role of lipoprotein lipase in adipose tissue development and metabolism. Int. J. Obes. Relat. Metab. Disord..

[64] Barchetta I., Cimini F.A., Chiappetta C., Bertoccini L., Ceccarelli V., Capoccia D., Gaggini M., Cristofano C.D., Rocca C.D., Silecchia G. (2020). Relationship between hepatic and systemic angiopoietin-like 3, hepatic Vitamin D receptor expression and NAFLD in obesity. Liver Int..

[65] García-Monzón C., Petrov P.D., Rey E., Marañón P., Del Pozo-Maroto E., Guzmán C., Rodriguez de Cia J., Casado-Collado A.J., Vargas-Castrillon J., Saez A. (2018). Angiopoietin-Like Protein 8 Is a Novel Vitamin D Receptor Target Gene Involved in Nonalcoholic Fatty Liver Pathogenesis. Am. J. Pathol..

[66] Zhang H., Shen Z., Lin Y., Zhang J., Zhang Y., Liu P., Zeng H., Yu M., Chen X., Ning L. (2020). Vitamin D receptor targets hepatocyte nuclear factor 4α and mediates protective effects of vitamin D in nonalcoholic fatty liver disease. J. Biol. Chem..

[67] Xu Y., Zalzala M., Xu J., Li Y., Yin L., Zhang Y. (2015). A metabolic stress-inducible miR-34a-HNF4α pathway regulates lipid and lipoprotein metabolism. Nat. Commun..

[68] Italian Association for the Study of the Liver (AISF) (2017). AISF position paper on nonalcoholic fatty liver disease (NAFLD): Updates and future directions. Dig. Liver Dis..

[69] Bashir A., Duseja A., De A., Mehta M., Tiwari P. (2022). Non-alcoholic fatty liver disease development: A multifactorial pathogenic phenomena. Liver Res..

[70] Utzschneider K.M., Kahn S.E. (2006). The Role of Insulin Resistance in Nonalcoholic Fatty Liver Disease. J. Clin. Endocrinol. Metab..

[71] Elhafiz M., Zhao G., Ismail M., Xu D., Das D., Fan S., Cheng N., Yousef B.A., Jiang Z., Zhang L. (2020). Imbalanced insulin substrate-1 and insulin substrate-2 signaling trigger hepatic steatosis in vitamin D deficient rats: 8-methoxypsoralen, a vitamin D receptor ligand with a promising anti-steatotic action. Biochim. Biophys. Acta Mol. Cell. Biol. Lipids.

[72] Dong B., Zhou Y., Wang W., Scott J., Kim K., Sun Z., Guo Q., Lu Y., Gonzales N.M., Wu H. (2020). Vitamin D Receptor Activation in Liver Macrophages Ameliorates Hepatic Inflammation, Steatosis, and Insulin Resistance in Mice. Hepatology.

[73] Oh J., Riek A.E., Darwech I., Funai K., Shao J., Chin K., Sierra O.L., Carmeliet G., Ostlund Jr R.E., Mizrachi C.B. (2015). Deletion of Macrophage Vitamin D Receptor Promotes Insulin Resistance and Monocyte Cholesterol Transport to Accelerate Atherosclerosis in Mice. Cell. Rep..

[74] Ogunkolade B.W., Boucher B.J., Prahl J.M., Bustin S.A., Burrin J.M., Noonan K., North B.V., Mannan N., McDermott M.F., DeLuca H.F. (2002). Vitamin D receptor (VDR) mRNA and VDR protein levels in relation to vitamin D status, insulin secretory capacity, and VDR genotype in Bangladeshi Asians. Diabetes.

[75] Su D., Nie Y., Zhu A., Chen Z., Wu P., Zhang L., Luo M., Sun Q., Cai Y., Xiao Z. (2016). Vitamin D Signaling through Induction of Paneth Cell Defensins Maintains Gut Microbiota and Improves Metabolic Disorders and Hepatic Steatosis in Animal Models. Front. Physiol..

[76] Pendyala S., Walker J.M., Holt P.R. (2012). A high-fat diet is associated with endotoxemia that originates from the gut. Gastroenterology.

[77] Cani P.D., Amar J., Iglesias M.A., Poggi M., Knauf C., Bastelica D., Neyrinck A.M., Fava F., Tuohy K.M., Chabo C. (2007). Metabolic endotoxemia initiates obesity and insulin resistance. Diabetes.

[78] Kolodziejczyk A.A., Zheng D., Shibolet O., Elinav E. (2019). The role of the microbiome in NAFLD and NASH. EMBO Mol. Med..

[79] Jin D., Wu S., Zhang Y.G., Lu R., Xia Y., Dong H., Sun J. (2015). Lack of Vitamin D Receptor Causes Dysbiosis and Changes the Functions of the Murine Intestinal Microbiome. Clin. Ther..

[80] Wu S., Zhang Y.G., Lu R., Xia Y., Zhou D., Petrof E.O., Claud E.C., Chen D., Chang E.B., Carmeliet G. (2015). Intestinal epithelial vitamin D receptor deletion leads to defective autophagy in colitis. Gut.

[81] Akimbekov N.S., Digel I., Sherelkhan D.K., Lutfor A.B., Razzaque M.S. (2020). Vitamin D and the Host-Gut Microbiome: A Brief Overview. Acta Histochem. Cytochem..

[82] Chatterjee I., Lu R., Zhang Y., Zhang J., Dai Y., Xia Y., Sun J. (2020). Vitamin D receptor promotes healthy microbial metabolites and microbiome. Sci. Rep..

[83] Musso G., Cassader M., Paschetta E., Gambino R. (2018). Bioactive Lipid Species and Metabolic Pathways in Progression and Resolution of Nonalcoholic Steatohepatitis. Gastroenterology.

[84] Neuschwander-Tetri B.A. (2010). Hepatic lipotoxicity and the pathogenesis of nonalcoholic steatohepatitis: The central role of nontriglyceride fatty acid metabolites. Hepatology.

[85] Ricca C., Aillon A., Bergandi L., Alotto D., Castagnoli C., Silvagno F. (2018). Vitamin D Receptor Is Necessary for Mitochondrial Function and Cell Health. Int. J. Mol. Sci..

[86] Leamy A.K., Egnatchik R.A., Young J.D. (2013). Molecular mechanisms and the role of saturated fatty acids in the progression of non-alcoholic fatty liver disease. Prog. Lipid Res..

[87] Malhi H., Kaufman R.J. (2011). Endoplasmic reticulum stress in liver disease. J. Hepatol..

[88] Zhou Y., Dong B., Kim K.H., Choi S., Sun Z., Wu N., Wu Y., Scott J., Moore D.D. (2020). Vitamin D Receptor Activation in Liver Macrophages Protects Against Hepatic Endoplasmic Reticulum Stress in Mice. Hepatology.

[89] Wen G., Eder K., Ringseis R. (2020). 1,25-hydroxyvitamin D3 decreases endoplasmic reticulum stress-induced inflammatory response in mammary epithelial cells. PLoS ONE.

[90] Roh Y.S., Seki E. (2013). Toll-like receptors in alcoholic liver disease, non-alcoholic steatohepatitis and carcinogenesis. J. Gastroenterol. Hepatol..

[91] Tomita K., Tamiya G., Ando S., Ohsumi K., Chiyo T., Mizutani A., Kitamura N., Toda K., Kaneko T., Horie Y. (2006). Tumour necrosis factor alpha signalling through activation of Kupffer cells plays an essential role in liver fibrosis of non-alcoholic steatohepatitis in mice. Gut.

[92] Fritsche J., Mondal K., Ehrnsperger A., Andreesen R., Kreutz M. (2003). Regulation of 25-hydroxyvitamin D3-1 alpha-hydroxylase and production of 1 alpha,25-dihydroxyvitamin D3 by human dendritic cells. Blood.

[93] Arora J., Wang J., Weaver V., Zhang Y., Cantorna M.T. (2022). Novel insight into the role of the vitamin D receptor in the development and function of the immune system. J. Steroid Biochem. Mol. Biol..

[94] Spittler A., Willheim M., Leutmezer F., Ohler R., Krugluger W., Reissner C., Luca T., Brodowicz T., Roth E., Boltz-Nitulescu G. (1997). Effects of 1 alpha,25-dihydroxyvitamin D3 and cytokines on the expression of MHC antigens, complement receptors and other antigens on human blood monocytes and U937 cells: Role in cell differentiation, activation and phagocytosis. Immunology.

[95] Rendra E., Riabov V., Mossel D.M., Sevastyanova T., Harmsen M.C., Kzhyshkowska J. (2019). Reactive oxygen species (ROS) in macrophage activation and function in diabetes. Immunobiology.

[96] Chen Y., Liu W., Sun T., Huang Y., Wang Y., Deb D.K., Yoon D., Kong J., Thadhani R., Chun Li Y. (2013). 1,25-Dihydroxyvitamin D promotes negative feedback regulation of TLR signaling via targeting microRNA-155-SOCS1 in macrophages. J. Immunol..

[97] Ellergezen P., Alp A., Çavun S. (2023). Vitamin D, VDR, and VDBP Levels Correlate with Anti-inflammatory Cytokine Profile in FMS Patients. Med. Rec..

[98] Affo S., Yu L.X., Schwabe R.F. (2017). The Role of Cancer-Associated Fibroblasts and Fibrosis in Liver Cancer. Annu. Rev. Pathol..

[99] Mihm S. (2018). Danger-Associated Molecular Patterns (DAMPs): Molecular Triggers for Sterile Inflammation in the Liver. Int. J. Mol. Sci..

[100] Watanabe A., Hashmi A., Gomes D.A., Town T., Badou A., Flavell R.A., Mehal W.Z. (2007). Apoptotic hepatocyte DNA inhibits hepatic stellate cell chemotaxis via toll-like receptor 9. Hepatology.

[101] Duran A., Hernandez E.D., Reina-Campos M., Castilla E.A., Subramaniam S., Raghunandan S., Roberts L.R., Kisseleva T., Karin M., Diaz-Meco M.T. (2016). p62/SQSTM1 by Binding to Vitamin D Receptor Inhibits Hepatic Stellate Cell Activity, Fibrosis, and Liver Cancer. Cancer Cell.

[102] Abramovitch S., Dahan-Bachar L., Sharvit E., Weisman Y., Tov B.A., Brazowski E., Reif S. (2011). Vitamin D inhibits proliferation and profibrotic marker expression in hepatic stellate cells and decreases thioacetamide-induced liver fibrosis in rats. Gut.

[103] Neeman R., Abramovitch S., Sharvit E., Elad-Sfadia G., Haklai R., Kloog Y., Reif S. (2014). Vitamin D and S-farnesylthiosalicylic acid have a synergistic effect on hepatic stellate cells proliferation. Dig. Dis. Sci..

[104] Roderfeld M. (2018). Matrix metalloproteinase functions in hepatic injury and fibrosis. Matrix Biol..

[105] Veidal S.S., Vassiliadis E., Barascuk N., Zhang C., Segovia-Silvestre T., Klickstein L., Larsen M.R., Qvist P., Christiansen C., Vainer B. (2010). Matrix metalloproteinase-9-mediated type III collagen degradation as a novel serological biochemical marker for liver fibrogenesis. Liver Int..

[106] Schuppan D., Surabattula R., Wang X.Y. (2018). Determinants of fibrosis progression and regression in NASH. J. Hepatol..

[107] Halder S.K., Osteen K.G., Al-Hendy A. (2013). Vitamin D3 inhibits expression and activities of matrix metalloproteinase-2 and -9 in human uterine fibroid cells. Hum. Reprod..

[108] Rahman A., Hershey S., Ahmed S., Nibbelink K., Simpson R.U. (2007). Heart extracellular matrix gene expression profile in the vitamin D receptor knockout mice. J. Steroid Biochem. Mol. Biol..

[109] Roehlen N., Crouchet E., Baumert T.F. (2020). Liver Fibrosis: Mechanistic Concepts and Therapeutic Perspectives. Cells.

[110] Saffioti F., Pinzani M. (2016). Development and Regression of Cirrhosis. Dig. Dis..

[111] Abramovitch S., Sharvit E., Weisman Y., Bentov A., Brazowski E., Cohen G., Volovelsky O., Reif S. (2015). Vitamin D inhibits development of liver fibrosis in an animal model but cannot ameliorate established cirrhosis. Am. J. Physiol. Gastrointest. Liver Physiol..

[112] Ying H.Z., Chen Q., Zhang W.Y., Zhang H.H., Ma Y., Zhang S.Z., Fang J., Yu C.H. (2017). PDGF signaling pathway in hepatic fibrosis pathogenesis and therapeutics. Mol. Med. Rep..

[113] Pedigo N., Zhang H., Koszewski N.J., Kaetzel D.M. (2003). A 5’-distal element mediates vitamin D-inducibility of PDGF-A gene transcription. Growth Factors.

[114] Walton K.L., Johnson K.E., Harrison C.A. (2017). Targeting TGF-β Mediated SMAD Signaling for the Prevention of Fibrosis. Front. Pharmacol..

[115] Ding N., Yu R.T., Subramaniam N., Sherman M.H., Wilson C., Rao R., Leblanc M., Coulter S., He M., Scott C. (2013). A vitamin D receptor/SMAD genomic circuit gates hepatic fibrotic response. Cell.

[116] Beilfuss A., Sowa J.P., Sydor S., Beste M., Bechmann L.P., Schlattjan M., Syn W.K., Wedemeyer I., Mathe Z., Jochum C. (2015). Vitamin D counteracts fibrogenic TGF-β signalling in human hepatic stellate cells both receptor-dependently and independently. Gut.

[117] Wang X., Wang G., Qu J., Yuan Z., Pan R., Li K. (2020). Calcipotriol Inhibits NLRP3 Signal Through YAP1 Activation to Alleviate Cholestatic Liver Injury and Fibrosis. Front. Pharmacol..

[118] Rao Z., Chen X., Wu J., Xiao M., Zhang J., Wang B., Fang L., Zhang H., Wang X., Yang S. (2019). Vitamin D Receptor Inhibits NLRP3 Activation by Impeding Its BRCC3-Mediated Deubiquitination. Front. Immunol..

[119] Harini K.S., Ezhilarasan D. (2022). Wnt/beta-catenin signaling and its modulators in nonalcoholic fatty liver diseases. Hepatobiliary Pancreat. Dis. Int..

[120] Lecarpentier Y., Schussler O., Hébert J.L., Vallée A. (2019). Multiple Targets of the Canonical WNT/β-Catenin Signaling in Cancers. Front. Oncol..

[121] Guo Y., Xiao L., Sun L., Liu F. (2012). Wnt/beta-catenin signaling: A promising new target for fibrosis diseases. Physiol. Res..

[122] Egan J.B., Thompson P.A., Vitanov M.V., Bartik L., Jacobs E.T., Haussler M.R., Gerner E.W., Jurutka P.W. (2010). Vitamin D receptor ligands, adenomatous polyposis coli, and the vitamin D receptor FokI polymorphism collectively modulate beta-catenin activity in colon cancer cells. Mol. Carcinog..

[123] Sari E., Oztay F., Tasci A.E. (2020). Vitamin D modulates E-cadherin turnover by regulating TGF-β and Wnt signalings during EMT-mediated myofibroblast differentiation in A459 cells. J. Steroid Biochem. Mol. Biol..

[124] Khan F.Z., Perumpail R.B., Wong R.J., Ahmed A. (2015). Advances in hepatocellular carcinoma: Nonalcoholic steatohepatitis-related hepatocellular carcinoma. World J. Hepatol..

[125] Dhar D., Baglieri J., Kisseleva T., Brenner D.A. (2020). Mechanisms of liver fibrosis and its role in liver cancer. Exp. Biol. Med. (Maywood).

[126] Baglieri J., Brenner D.A., Kisseleva T. (2019). The Role of Fibrosis and Liver-Associated Fibroblasts in the Pathogenesis of Hepatocellular Carcinoma. Int. J. Mol. Sci..

[127] Kong F., Li L., Wang G., Deng X., Li Z., Kong X. (2019). VDR signaling inhibits cancer-associated-fibroblasts’ release of exosomal miR-10a-5p and limits their supportive effects on pancreatic cancer cells. Gut.

[128] Ferrer-Mayorga G., Gómez-López G., Barbáchano A., Fernández-Barral A., Peña C., Pisano D.G., Camtero R., Rojo F., Munoz A., Larriba M.J. (2017). Vitamin D receptor expression and associated gene signature in tumour stromal fibroblasts predict clinical outcome in colorectal cancer. Gut.

[129] Campos L.T., Brentani H., Roela R.A., Katayama M.L.H., Lima L., Rolim C.F., Milani C., Azevedo Koike Folgueira M.A., Brentani M.M. (2013). Differences in transcriptional effects of 1α,25 dihydroxyvitamin D3 on fibroblasts associated to breast carcinomas and from paired normal breast tissues. J. Steroid Biochem. Mol. Biol..

[130] Zhao Z.X., Zhang Y.Q., Sun H., Chen Z.Q., Chang J.J., Wang X., Wang X., Tan C., Ni S.J., Weng W.W. (2023). Calcipotriol abrogates cancer-associated fibroblast-derived IL-8-mediated oxaliplatin resistance in gastric cancer cells via blocking PI3K/Akt signaling. Acta Pharmacol. Sin..

[131] Giulianelli S., Cerliani J.P., Lamb C.A., Fabris V.T., Bottino M.C., Gorostiaga M.A., Novaro V., Gongora A., Baldi A., Molinolo A. (2008). Carcinoma-associated fibroblasts activate progesterone receptors and induce hormone independent mammary tumor growth: A role for the FGF-2/FGFR-2 axis. Int. J. Cancer.

[132] Peña C., Céspedes M.V., Lindh M.B., Kiflemariam S., Mezheyeuski A., Edqvist P.H., Hagglof C., Birgisson H., Bojmar L., Jirstrom K. (2013). STC1 expression by cancer-associated fibroblasts drives metastasis of colorectal cancer. Cancer Res..

[133] Sewell-Loftin M.K., Bayer S.V.H., Crist E., Hughes T., Joison S.M., Longmore G.D., George S.C. (2017). Cancer-associated fibroblasts support vascular growth through mechanical force. Sci. Rep..

[134] Zheng Q., Martin R.C., Shi X., Pandit H., Yu Y., Liu X., Guo W., Tan M., Bai Q., Meng X. (2020). Lack of FGF21 promotes NASH-HCC transition via hepatocyte-TLR4-IL-17A signaling. Theranostics.

[135] Gomes A.L., Teijeiro A., Burén S., Tummala K.S., Yilmaz M., Waisman A., Theurillat J.P., Perma C., Djouder N. (2016). Metabolic Inflammation-Associated IL-17A Causes Non-alcoholic Steatohepatitis and Hepatocellular Carcinoma. Cancer Cell.

[136] Tezze C., Romanello V., Sandri M. (2019). FGF21 as Modulator of Metabolism in Health and Disease. Front Physiol..

[137] Wang D., Huang H.J., Kazlauskas A., Cavenee W.K. (1999). Induction of vascular endothelial growth factor expression in endothelial cells by platelet-derived growth factor through the activation of phosphatidylinositol 3-kinase. Cancer Res..

[138] Jamali N., Song Y.S., Sorenson C.M., Sheibani N. (2019). 1,25(OH)2D3 regulates the proangiogenic activity of pericyte through VDR-mediated modulation of VEGF production and signaling of VEGF and PDGF receptors. FASEB Bioadv.

[139] Kim R., Emi M., Tanabe K. (2007). Cancer immunoediting from immune surveillance to immune escape. Immunology.

[140] Mougiakakos D., Choudhury A., Lladser A., Kiessling R., Johansson C.C. (2010). Regulatory T cells in cancer. Adv. Cancer Res..

[141] Ostrand-Rosenberg S. (2010). Myeloid-derived suppressor cells: More mechanisms for inhibiting antitumor immunity. Cancer Immunol. Immunother..

[142] Monteran L., Erez N. (2019). The Dark Side of Fibroblasts: Cancer-Associated Fibroblasts as Mediators of Immunosuppression in the Tumor Microenvironment. Front. Immunol..

[143] Li P., Zhu X., Cao G., Wu R., Li K., Yuan W., Chen B., Sun G., Xia X., Zhang H. (2022). 1α,25(OH)2D3 reverses exhaustion and enhances antitumor immunity of human cytotoxic T cells. J. Immunother. Cancer.

[144] Bochen F., Balensiefer B., Körner S., Bittenbring J.T., Neumann F., Koch A., Bumm K., Marx A., Wemmert S., Papaspyrou G. (2018). Vitamin D deficiency in head and neck cancer patients–prevalence, prognostic value and impact on immune function. Oncoimmunology.

[145] Hutchinson P.E., Pringle J.H. (2022). Consideration of possible effects of vitamin D on established cancer, with reference to malignant melanoma. Pigment. Cell. Melanoma Res..

[146] Chen Y., Zhang J., Ge X., Du J., Deb D.K., Li Y.C. (2013). Vitamin D receptor inhibits nuclear factor κB activation by interacting with IκB kinase β protein. J. Biol. Chem..

[147] Liu T., Zhang L., Joo D., Sun S.C. (2017). NF-κB signaling in inflammation. Signal. Transduct. Target. Ther..

[148] Guo J., Ma Z., Ma Q., Wu Z., Fan P., Zhou X., Chen L., Zhou S., Goltzman D., Miao D. (2013). 1, 25(OH)2D3 Inhibits Hepatocellular Carcinoma Development Through Reducing Secretion of Inflammatory Cytokines from Immunocytes. Curr. Med. Chem..

[149] Bishop L.E., Ismailova A., Dimeloe S., Hewison M., White J.H. (2021). Vitamin D and Immune Regulation: Antibacterial, Antiviral, Anti-Inflammatory. JBMR Plus.

[150] von Essen M.R., Kongsbak M., Schjerling P., Olgaard K., Odum N., Geisler C. (2010). Vitamin D controls T cell antigen receptor signaling and activation of human T cells. Nat. Immunol..

[151] Lemire J.M. (1992). Immunomodulatory role of 1,25-dihydroxyvitamin D3. J. Cell. Biochem..

[152] Scolletta S., Colletti M., Luigi L.D., Crescioli C. (2013). Vitamin D receptor agonists target CXCL10, New therapeutic tools for resolution of inflammation. Mediat. Inflamm..

[153] Boonstra A., Barrat F.J., Crain C., Heath V.L., Savelkoul H.F., O’Garra A. (2001). 1alpha,25-Dihydroxyvitamin d3 has a direct effect on naive CD4(+) T cells to enhance the development of Th2 cells. J. Immunol..

[154] Penna G., Roncari A., Amuchastegui S., Daniel K.C., Berti E., Colonna M., Adorini L. (2005). Expression of the inhibitory receptor ILT3 on dendritic cells is dispensable for induction of CD4+Foxp3+ regulatory T cells by 1,25-dihydroxyvitamin D3. Blood.

[155] Kim Y., Chang Y., Ryu S., Cho I.Y., Kwon M.J., Sohn W., Kim M.K., Wild S.H., Byrne C.D. (2022). Resolution of, and Risk of Incident Non-alcoholic Fatty Liver Disease With Changes in Serum 25-hydroxy Vitamin D Status. J. Clin. Endocrinol. Metab..

[156] Chen Y., Feng S., Chang Z., Zhao Y., Liu Y., Fu J., Liu Y., Tang S., Han Y., Zhang S. (2022). Higher Serum 25-Hydroxyvitamin D Is Associated with Lower All-Cause and Cardiovascular Mortality among US Adults with Nonalcoholic Fatty Liver Disease. Nutrients.

[157] Gong J., Gong H., Liu Y., Tao X., Zhang H. (2022). Calcipotriol attenuates liver fibrosis through the inhibition of vitamin D receptor-mediated NF-κB signaling pathway. Bioengineered.

[158] Dalhoff K., Dancey J., Astrup L., Skovsgaard T., Hamberg K.J., Lofts F.J., Rosmorduc O., Erlinger S., Bach Hansen J., Steward W.P. (2003). A phase II study of the vitamin D analogue Seocalcitol in patients with inoperable hepatocellular carcinoma. Br. J. Cancer.

